# Transplanted human fecal microbiota enhanced Guillain Barré syndrome autoantibody responses after *Campylobacter jejuni* infection in C57BL/6 mice

**DOI:** 10.1186/s40168-017-0284-4

**Published:** 2017-08-08

**Authors:** Phillip T. Brooks, Kelsey A. Brakel, Julia A. Bell, Christopher E. Bejcek, Trey Gilpin, Jean M. Brudvig, Linda S. Mansfield

**Affiliations:** 1 Comparative Enteric Diseases Laboratory, Michigan State University, East Lansing, MI USA; 2 Comparative Medicine and Integrative Biology, Michigan State University, East Lansing, MI USA; 3 Institute for Integrative Toxicology, Michigan State University, East Lansing, MI USA; 4 College of Veterinary Medicine, Michigan State University, East Lansing, MI USA; 5 Department of Microbiology and Molecular Genetics, Michigan State University, 181 Food Safety Building; 1129 Farm Lane, East Lansing, MI 48824 USA

**Keywords:** *Campylobacter jejuni*, Guillain-Barré syndrome, Commensal microbiota, Mouse models, Autoimmunity, Gastrointestinal inflammation, Broad-spectrum antibiotics

## Abstract

**Background:**

*Campylobacter jejuni* is the leading antecedent infection to the autoimmune neuropathy Guillain-Barré syndrome (GBS), which is accompanied by an autoimmune anti-ganglioside antibody attack on peripheral nerves. Previously, we showed that contrasting immune responses mediate *C. jejuni* induced colitis and autoimmunity in interleukin-10 (IL-10)-deficient mice, dependent upon the infecting strain. Strains from colitis patients elicited T helper 1 (T_H_1)-dependent inflammatory responses while strains from GBS patients elicited T_H_2-dependent autoantibody production. Both syndromes were exacerbated by antibiotic depletion of the microbiota, but other factors controlling susceptibility to GBS are unknown.

**Methods:**

Using 16S rRNA gene high-throughput sequencing, we examined whether structure of the gut microbial community alters host (1) gastrointestinal inflammation or (2) anti-ganglioside antibody responses after infection with *C. jejuni* strains from colitis or GBS patients. We compared these responses in C57BL/6 mice with either (1) stable human gut microbiota (^Hu^microbiota) transplants or (2) conventional mouse microbiota (^Conv^microbiota).

**Results:**

Inoculating germ-free C57BL/6 wild-type (WT) mice with a mixed human fecal slurry provided a murine model that stably passed its microbiota over >20 generations. Mice were housed in specific pathogen-free (SPF) facilities, while extra precautions of having caretakers wear sterile garb along with limited access ensured that no mouse pathogens were acquired. ^Hu^microbiota conferred many changes upon the WT model in contrast to previous results, which showed only colonization with no disease after *C. jejuni* challenge. When compared to ^Conv^microbiota mice for susceptibility to *C. jejuni* enteric or GBS patient strains, infected ^Hu^microbiota mice had (1) 10-100 fold increases in *C. jejuni* colonization of both strains, (2) pathologic change in draining lymph nodes but only mild changes in colon or cecal lamina propria, (3) significantly lower Th1/Th17-dependent anti-*C. jejuni* responses, (4) significantly higher IL-4 responses at 5 but not 7 weeks post infection (PI), (5) significantly higher Th2-dependent anti-*C. jejuni* responses, and (6) significantly elevated anti-ganglioside autoantibodies after *C. jejuni* infection. These responses in ^Hu^microbiota mice were correlated with a dominant *Bacteroidetes* and *Firmicutes* microbiota.

**Conclusions:**

These data demonstrate that ^Hu^microbiota altered host-pathogen interactions in infected mice, increasing colonization and Th-2 and autoimmune responses in a *C. jejuni* strain-dependent manner. Thus, microbiota composition is another factor controlling susceptibility to GBS.

**Electronic supplementary material:**

The online version of this article (doi:10.1186/s40168-017-0284-4) contains supplementary material, which is available to authorized users.

## Background

Dysbiosis, the depletion of beneficial organisms in the gut microbiota, has been implicated in the manifestation of several autoimmune and chronic inflammatory diseases. Both autoimmune diseases and chronic inflammatory diseases are characterized by destruction of tissues and functional impairment modulated by immune mechanisms [1–3]. The microbiota modulates host immune responses and affects the production of cytokines, antibodies, and antimicrobial peptides that target pathogens for removal [4, 5]. Microbial regulation of these immune responses highlights the importance of host-microbiota mutualism. Thus, several autoimmune diseases likely have origins in dysbiotic microbiota or abnormal mucosal barrier function including inflammatory bowel disease (IBD) [6], type 1 diabetes [7], multiple sclerosis [8, 9], and Reiter’s arthritis [10, 11]. A substantial number of autoimmune diseases including Guillain-Barré syndrome [12], Miller-Fisher syndrome [13], and Lyme arthritis [14] have been linked to previous infection with pathogenic organisms [15]. Considerable effort has been made to understand how infection with pathogenic microorganisms results in a loss of tolerance and initiates autoimmunity [15, 16]. One leading hypothesis is that molecular mimicry, a similarity between molecular structures on the infectious agent and host tissues, results in cross-reactivity [17, 18], which in turn leads to autoimmune attack.

*Campylobacter jejuni*, a leading cause of bacterial gastroenteritis worldwide, precedes at least one-fourth of all cases of the acute peripheral neuropathy Guillain-Barré syndrome [19–21]. It is hypothesized that molecular mimicry of host nerve gangliosides such as GM1a, GD1a, and GQ1b by the outer core of lipooligosaccharides on the surface of *C. jejuni* initiates cross-reactive antibody responses resulting in complement-mediated nerve damage [22]. Other factors may also contribute to Guillain-Barré syndrome (GBS) disease. When enteric disease is severe, *C. jejuni* infection may be treated with fluoroquinolone or macrolide antibiotics; however, increasing resistance to these drugs has been reported [23]. Notably, antibiotic-treated and gnotobiotic mice display increased susceptibility to *C. jejuni* colonization and enhanced incidence of gastrointestinal inflammation [24–26], leading to the hypothesis that components of the resident mouse gut microbiota protect against *Campylobacter-*mediated disease [26]. Moreover, approximately two-thirds of GBS patients report gastrointestinal or respiratory inflammation in the weeks preceding neurological symptoms [13, 27–29]; thus, host determinants of inflammation including gut microbiota may play a critical role in GBS development. Experimentally, normal flora of mice play a decisive role in preventing *C. jejuni-*mediated inflammation, thus raising the question of whether murine models carrying certain human microbiota would show similar susceptibility to enteric disease shown in depleted microbiota models [24–26].

C57BL/6 IL-10^+/+^ and IL-10^−/−^ mice function as *C. jejuni* colonization and colitis models, respectively [30]. C57BL/6 IL-10^−/−^ mice orally infected with isolates from patients with colitis had significantly upregulated type 1 and 17 but not type 2 cytokines in the colon coincident with infiltration of phagocytes, T cells, and innate lymphoid cells (ILCs) [31]. Anti-*C. jejuni* antibodies generated in this response were of different isotypes; type 1 responses produced IgG2c antibodies, while type 17 responses produced IgG2b antibodies. However, *C. jejuni* strains from GBS patients induced mild colitis in C57BL/6 IL-10^−/−^ mice associated with blunted type 1/17 but enhanced type 2 responses. Only type 2 antibodies cross-reacted with nerve gangliosides reflecting the roles of Th1/17 responses in killing intracellular pathogens and of Th2 responses in the induction of autoimmunity [31]. These type 2 antibodies were of the IgG1 isotype. We chose the C57BL/6 model to examine the role of the microbiome in eliciting autoimmunity.

To determine if a humanized microbial community is sufficient to alter the host inflammatory and autoimmune response to infection with *C. jejuni*, we infected C57BL/6 humanized (^Hu^microbiota) and conventional microbiota (^Conv^microbiota) colonized mice with a *C. jejuni* enteric disease patient strain (11168) or GBS patient strain (260.94). Using an established and robust experimental inoculation system and a defined set of disease indicators [30–32], we measured both inflammatory and autoimmune endpoints. We hypothesized that ^Hu^microbiota mice would exhibit (1) enhanced colonization by *C. jejuni*, (2) higher levels of anti-ganglioside antibodies, and (3) increased lesions in both the GI tract and peripheral nerves compared to mice with ^Conv^microbiota. To compare the microbiota of ^Hu^microbiota and ^Conv^microbiota mice, we characterized the fecal microbiota using 16S rRNA gene amplicon analysis. To examine whether the expected microbiota-dependent immune responses resulted in inflammatory changes in the gut (assessed by gross pathology, histopathology, and colon homogenate IFNγ and IL-4 levels) or elevated anti-ganglioside autoantibody levels (determined by plasma antibody ELISA), we infected both ^Hu^microbiota and ^Conv^microbiota mice with *C. jejuni* 11168 and *C. jejuni* 260.94. Here, we show that infected ^Hu^microbiota mice had significantly higher levels of *C. jejuni* colonization, demonstrable clinical signs of *C. jejuni* gastroenteritis, and shifts in their adaptive immune responses toward a type 2 biased antibody response with significantly elevated anti-ganglioside autoantibodies when compared to ^Conv^microbiota mice. These outcomes were affected by the characteristics of the infecting *C. jejuni* strain as well as by the composition of the gut microbiota. Interestingly, ^Hu^microbiota mice also had diminished activity in the open-field test that was not associated with infection status.

## Results

### Overview

Inoculating germ-free C57BL/6 wild-type (WT) mice with a mixed human fecal slurry provided a murine model that stably passed its microbiota over >20 generations. In two separate experiments (Pilot, Experiment 1), we show that this ^Hu^microbiota conferred many changes upon the WT model that previously showed only colonization with no disease after *C. jejuni* challenge. ^Hu^microbiota mice infected with either *C. jejuni* 11168 from a patient with enteritis or *C. jejuni* 260.94 from a patient with GBS had significant increases in colonization levels compared to infected ^Conv^microbiota mice. These two groups of infected ^Hu^microbiota mice also had pathologic changes in draining lymph nodes, and colon and cecal lamina propria that were not seen in infected ^Conv^microbiota mice. The immunologic responses to *C. jejuni* were also altered in infected ^Hu^microbiota mice with significantly lower Th1/Th17-dependent and higher Th2-dependent anti-*C. jejuni* responses. The presence of higher Th2-dependent anti-*C. jejuni* responses were correlated with significantly elevated anti-ganglioside autoantibodies after *C. jejuni* infection. These responses in ^Hu^microbiota mice were correlated with a dominant *Bacteroidetes* and *Firmicutes* microbiota. Both experiments were conducted similarly except that mice were euthanized and necropsied 5 weeks after *C. jejuni* inoculation in the pilot experiment and 7 weeks after *C. jejuni* inoculation in Experiment 1.

## Pilot experiment

### Fecal microbiota

To determine if handling (SPF v sterile) altered the microbiota of ^Hu^microbiota mice, we compared the fecal microbiota of ^Hu^microbiota and ^Conv^microbiota mice by qPCR. For this analysis, we amplified fecal DNA using 16S rRNA gene primers specific for *Clostridium* group 14, *Clostridium* group 1, *Bacteroidetes*, and *Enterobacteriaceae*. No statistically significant differences were detected in these four bacterial groups between ^Hu^microbiota mice sham inoculated with tryptose soya broth (TSB) kept under specific pathogen-free (SPF) conditions and ^Hu^microbiota mice sham inoculated with TSB kept under sterile conditions (Additional file 1: Figure S1).

### Disease indicators

**Fig. 1:**
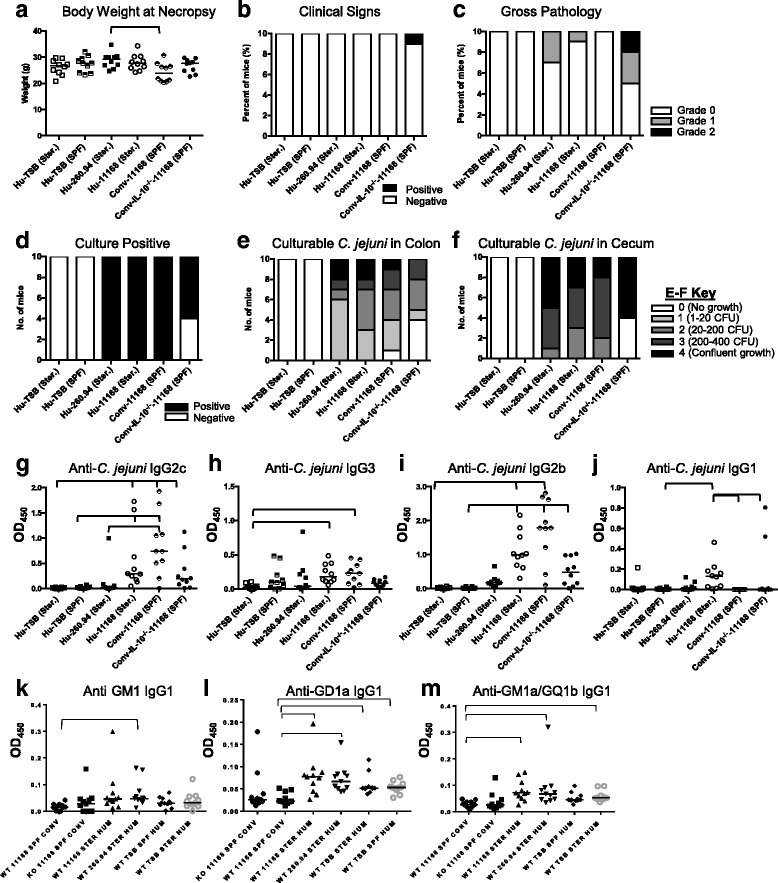
Disease indicators: Pilot experiment. (**a**) body weight at necropsy, (**b**) clinical signs, (**c**) gross pathology at necropsy, (**d**) number of mice that are culture positive for *C. jejuni* in cecum or colon, and semi-quantitative representation of culturable *C. jejuni* in (**e**) colon and (**f**) cecum at necropsy. Panels **g**-**m** represent anti-*Campylobacter* (**g**-**j**) and anti-ganglioside antibodies (**k**-**m**) detected by indirect ELISA. Bars indicate statistical significance. Data were analyzed by Kruskal-Wallis test on ranks and Dunn’s post-test where appropriate; p≤0.05 considered statistically significant. The microbiota type of the mouse is indicated as Hu (Human microbiota) or Conv (Conventional microbiota) followed by their treatment group

To determine if ^Hu^microbiota mice were (1) susceptible to *C. jejuni* gastroenteritis and (2) developed *C. jejuni* strain-specific antibody responses to GBS patient strains in the Pilot Experiment, we infected mice with either strain 11168 from an enteritis patient or strain 260.94 from a GBS patient or TSB sham inoculated them with the vehicle (Table 1). Veterinarians and trained animal handlers monitored mice for clinical signs daily. A significant difference in body weight was detected between Hu-260.94 and Conv-11168 mice at the time of necropsy (Fig. 1a). A single interleukin-10 (IL-10)-deficient infected mouse with conventional microbiota (Conv-IL-10^−/−^-11168 (SPF)) displayed severe clinical signs (Fig. 1b). Diarrhea on fur and rough hair coat are the clinical signs most often detected. Gross pathology was mild in all cases, infrequent with the exception of Conv-IL-10^−/−^-11168 mice, and restricted to infected mice in all cases (Fig. 1c). Interestingly, other than the IL-10-deficient mice, only infected ^Hu^microbiota mice showed gross pathology including thickened cecal and colon wall and enlarged ileocecocolic lymph nodes. *C. jejuni* 11168 infected C57BL/6 genetically wild-type and IL-10^−/−^ were used as gastroenteritis controls, due to their well-characterized reputation as colitis resistant (wild-type) and colitis susceptible (IL-10^−/−^) [30, 31]. *C. jejuni* could be cultured from mice in all infected groups (Fig. 1d–f); however, *C. jejuni* could be cultured from only 6 of 10 IL-10^−/−^ mice at necropsy. All IL-10^−/−^ mice had *C. jejuni*-positive fecal samples by culture at semi-quantitative levels of 3 or 4, on day 17 after inoculation showing that all experienced significant colonization. In comparison, 100% colonization at necropsy was achieved in *C. jejuni*-infected ^Hu^microbiota and ^Conv^microbiota wild-type (WT) mice, although the WT and IL-10^−/−^ mice were both derived from the same source (The Jackson Laboratory, Bar Harbor, MA).

### Mice with ^Hu^microbiota had greater T_H_2-dependent IgG1 responses to the enteric strain of *C. jejuni*, but lower such responses to the GBS strain

To assess specific immune reactivity and cross-reactive antibody responses, we measured anti-*C. jejuni* and anti-ganglioside antibody responses in infected mice by indirect enzyme-linked immunosorbent assay (ELISA). Presence of a human microbiota altered the response to the *C. jejuni* 11168 enteritis strain. Anti-*C. jejuni* IgG2c (T_H_1 associated) and IgG2b (T_H_17 associated) antibodies to 11168 were elevated compared to the TSB sham inoculated controls in ^Hu^microbiota C57BL/6 mice (Fig. 1g, i). However, the degree of elevation of both of these antibody isotypes was significantly lower in ^Hu^microbiota mice than in ^Conv^microbiota mice given the same *C. jejuni* strain (Fig. 1g, i). Furthermore, ^Hu^microbiota mice given *C. jejuni* 11168 mounted a significantly higher anti-*Campylobacter* T helper 2 (T_H_2)-biased IgG1 response, which was virtually absent in ^Conv^microbiota mice (Fig. 1j). Thus, having this human microbiota was sufficient to skew T helper cell responses to the enteric strain of *C. jejuni* toward an antibody-mediated adaptive response. Some ^Hu^microbiota mice infected with the *C. jejuni* 260.94 GBS strain had mild elevations in mixed T_H_1,/T_H_17/T_H_2 antibody responses, but these were not significant when compared to uninfected controls (Fig. 1g–j).

### Mice with ^Hu^microbiota had greater anti-ganglioside autoantibody responses than mice with ^Conv^microbiota

Presence of this human microbiota was sufficient to stimulate anti-ganglioside autoantibodies against the *C. jejuni* 11168 enteric strain that has not been previously associated with development of GBS. There were significantly increased anti-GD1a and anti-GM1a/GQ1b IgG1 autoantibodies in ^Hu^microbiota mice infected with the *C. jejuni* 11168 enteric strain compared to ^Conv^microbiota mice given the same strain (Fig. 1l, m). Also, despite the low anti-*C. jejuni* IgG1 antibody responses to the *C. jejuni* 260.94 strain, the IgG1-dependent anti-ganglioside antibody responses (GM1, GD1a, GM1/GQ1b) to the GBS strain 260.94 were significantly elevated in ^Hu^microbiota mice compared to ^Conv^microbiota mice given strain 11168 (Fig. 1k–m). Thus, infection with either of *C. jejuni* strains 260.94 or 11168 elicited anti-ganglioside autoantibodies, but only in ^Hu^microbiota mice. T_H_2-associated (IgG1) anti-ganglioside responses were elevated in some cases independent of inoculation status, suggesting a T_H_2-associated antibody bias in ^Hu^microbiota mice (Fig. 1l, m). This is reflected in the responses seen in TSB sham-inoculated uninfected control mice managed by either sterile or SPF techniques (Fig. 1l, m). This suggests that other pathogen-associated molecular patterns (PAMPs) from the microbiota may also cause ganglioside mimicry.

## Experiment 1

### ^Hu^microbiota mice have a distinct microbiota compared to ^Conv^microbiota mice

**Fig. 2:**
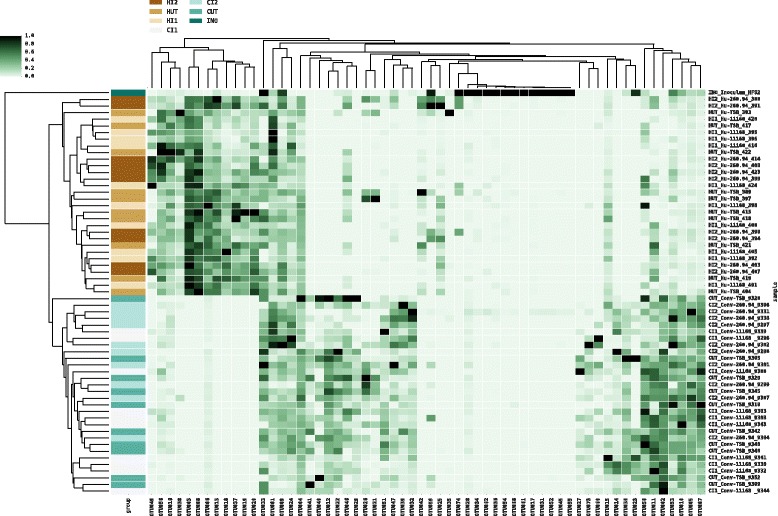
Heat map of relative OTU abundance across samples. Abundances were measured as proportions of samples and the 60 most abundant OTUs are shown. Samples and OTUs were clustered hierarchically based on relative abundance profiles. On the right y-axis labels represent individual samples starting with group labels. Group labels; HI2= Humanized-Infected-260.94, HUT= Humanized-Uninfected-TSB, HI1= Humanized-Infected-11168, CI2= Conventional-Infected-260.94, CUT= Conventional-Uninfected-TSB, CI1= Conventional-Infected-11168, INO= Inoculum. The left y-axis represents the color-coded groups shown in the legend. OTUs are represented on the x-axis with corresponding relative abundances shown in the heatmap grid with increasing abundance from light green to black

In Experiment 1 (Table 2), to compare microbiota structure in ^Hu^microbiota and ^Conv^microbiota, infected and TSB sham inoculated mice, we analyzed their fecal microbiota with 16S rRNA gene amplicon analysis. Analysis revealed that 4 of 5 phyla, 9 of 11 classes and 32 of 52 genera detected in the human fecal slurry used to produce the ^Hu^microbiota mice could be detected in the ^Hu^microbiota mice utilized in this study. The phylum *Verrucomicrobia* constituted less than 2% of the reads from the original inoculum and could not be found in ^Hu^microbiota mice used in this experiment. Noteworthy was that clustering of groups based on Bray-Curtis dissimilarity statistic resulted in separation by microbiota but not group assignments according to *C. jejuni* or TSB sham inoculation (Fig. 2).

**Table 1:** Experimental design: pilot experiment. C57BL/6 wild-type (C57BL/6) or congenic C57BL/6 IL-10-deficient (C57BL/6 IL-10^−/−^) mice with humanized (Hu) or conventional (Conv) microbiota were inoculated with TSB, *C. jejuni* 260.94, or *C. jejuni* 11168 and subjected to sterile (Ster.) or specific pathogen-free (SPF) handling for the duration of the experiment; 5 weeks post-inoculation

Group	Handling	Genotype	Microbiota	Inoculum	# of mice
Hu-TSB (Ster.)	Sterile	C57BL/6	Humanized	TSB	10
Hu-TSB (SPF)	SPF	C57BL/6	Humanized	TSB	10
Hu-260.94	Sterile	C57BL/6	Humanized	260.94	10
Hu-11168	Sterile	C57BL/6	Humanized	11168	10
Conv-11168	SPF	C57BL/6	Conventional	11168	10
Conv-IL-10^−/−^11168	SPF	C57BL/6 IL-10^−/−^	Conventional	11168	10

**Table 2:** Experimental Design: Experiment 1. C57BL/6 genetically wild-type mice with humanized (Hu) or conventional (Conv) microbiota were inoculated with TSB, *C. jejuni* 260.94, or *C. jejuni* 11168 and subjected to specific pathogen-free (SPF) handling for the duration of the experiment; 7 weeks post-inoculation

Group	Genotype	Microbiota	Inoculum	# of mice
Hu-TSB	C57BL/6	Humanized	TSB	10
Hu-260.94	C57BL/6	Humanized	*C. jejuni* 260.94	10
Hu-11168	C57BL/6	Humanized	*C. jejuni* 11168	10
Conv-TSB	C57BL/6	Murine	TSB	10
Conv-260.94	C57BL/6	Murine	*C. jejuni* 260.94	10
Conv-11168	C57BL/6	Murine	*C. jejuni* 11168	10

### There was an increased abundance of *Lactobacillus* in ^Conv^microbiota mice

**Fig. 3:**
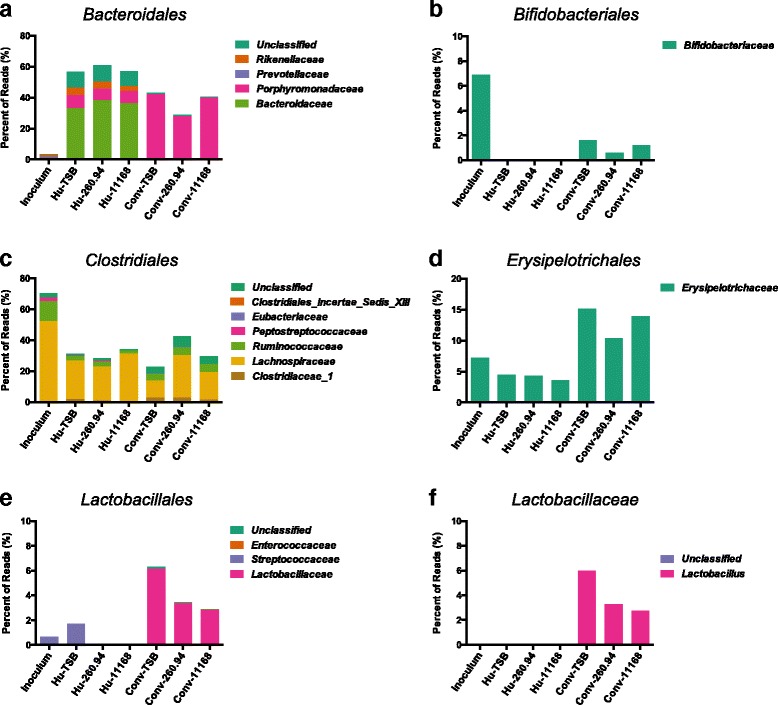
Relative abundance of major bacterial orders in fecal microbiota (**a**-**f**). Data represent relative abundances of OTUs assigned at the Order level with the exception of the family *Lactobacillaceae*. Orders constituting ≥5% of the average abundance for a single group were included. The average percentage of reads within each order that were assigned to families are represented as proportions of the orders bar

Five bacterial orders had an average abundance of 5% or greater in one or more groups of mice; *Bacteriodales*, *Bifidobacteriodales*, *Clostridiales*, *Lactobacillales*, and *Erysipelotrichales* (Fig. 3a–e). *Bacteriodales* was a minor component of the inoculum, constituting approximately 3.3% of the sequences, but a major component of ^Hu^microbiota (~57–60%) and ^Conv^microbiota (28–43%) (Fig. 3a). Within the order *Bacteriodales*, the ^Hu^microbiota was dominated by *Bacteroidaceae* (~58–63% of *Bacteriodales*) yet it was only a minor component of the conventional murine microbiota (~0.02–0.07% of *Bacteriodales*). In contrast, within the order *Bacteriodales*, the murine ^Conv^microbiota was dominated by *Porphyromonadaceae* (~98% of *Bacteriodales*). *Bifidobacteriodales* constituted ~7% of the inoculum, was completely absent in the ^Hu^microbiota mouse samples, and was a minor component of the ^Conv^microbiota (~0.6–1.6%) (Fig. 3b). All reads from the order *Bifidobacteriodales* were assigned to the family *Bifidobacteriaceae*. The inoculum was dominated by the order *Clostridiales* (~70%), which was less abundant in all of the mouse fecal samples but present in similar abundances in ^Hu^microbiota (~28–38%) and ^Conv^microbiota (22–42%) samples (Fig. 3c). Within the order *Clostridiales*, *Lachnospiraceae* was the dominating family in all groups. *Erysipelotrichales* was also present in all mice and was more abundant in ^Conv^microbiota mice than in the inoculum or ^Hu^microbiota mouse samples; inoculum (7.2%), ^Hu^microbiota (~3.6–4.5%), and ^Conv^microbiota (~10.4–15.1%) (Fig. 3d). *Lactobacillales* was present in all groups but more abundant in the ^Conv^microbiota mice than in the inoculum or in ^Hu^microbiota mice; inoculum (0.68%), ^Hu^microbiota (~0.002–1.7), and ^Conv^microbiota (~2.9–6.3%) (Fig. 3e). Within the order *Lactobacillales*, ^Hu^microbiota mice had no or 6000-fold less *Lactobacillaceae* than ^Conv^microbiota mouse samples. In all cases, greater than 97% of the reads in the family *Lactobacillaceae* were assigned to the genus *Lactobacillus* (Fig. 3f). At the order level, unclassified reads could be found in all groups; inoculum (3.7%), ^Hu^microbiota (~1.9–2.4%), and ^Conv^microbiota (~9.2–10.4%) (data not shown).

### Diversity statistics showed that groups can be distinguished by microbiota but not infection status

**Fig. 4:**
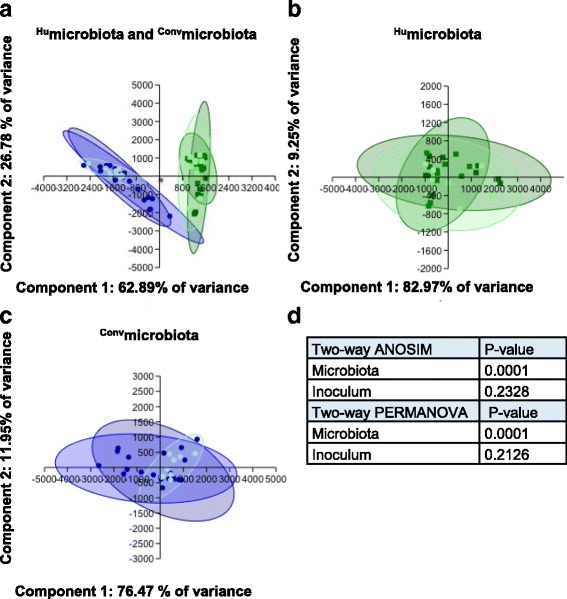
Principal component analysis (*PCA*) and multivariate statistics of 16S rRNA taxonomy. PCA modeling was performed using OTU assignments. Resulting plots show separation by microbiota (**a**) but not inoculum (**b** and **c**). Dots represent; dark blue = Conv-11168, blue = Conv-260.94, light blue = Conv-TSB, dark green = Hu-11168, green = Hu-260.94, light green = Hu-TSB, and red = Inoculum. **d** Two-way ANOSIM and two-way PERMANOVA indicate statistically significant differences between microbiota but not the inoculum

**Fig. 5:**
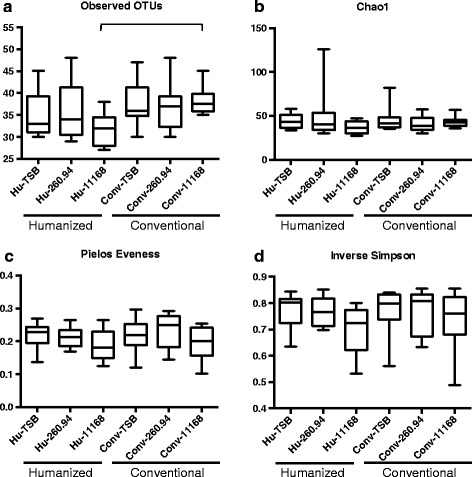
Alpha-diversity indices for 16S rRNA gene sequences. Panels represent (**a**) observed OTU’s, (**b**) estimated richness (Chao1), (**c**) species evenness (Pielos), and (**d**) species diversity (inverse Simpson). Data were analyzed by Kruskal-Wallis test on ranks and Dunn’s post-test; P≤0.05 was considered statistically significant. Whiskers represent minimum and maximum values. All other points are contained within the box and the bar represents the median

To determine if operational taxonomic units (OTUs) could be separated into groups based on microbiota or inoculation status, we assessed OTUs by alpha and beta diversity metrics. Principal components analysis showed that there was clear separation between ^Hu^microbiota and ^Conv^microbiota fecal samples (Fig. 4a) but that microbiota composition was not affected by inoculation status (Fig. 4b, c). These results are supported by two-way ANOSIM and PERMANOVA (Fig. 4d). Comparison of alpha diversity metrics revealed a disparity in OTUs in Hu-11168 compared to Conv-11168 (*P* = 0.0310) (Fig. 5a). No other disparity in alpha diversity of OTUs existed, and this finding was not reflected in species diversity or evenness (Fig. 5b–d).

### The majority of the variance between TSB sham-inoculated ^Hu^microbiota and ^Conv^microbiota mice can be attributed to the abundance of *Porphyromonadaceae*, *Bacteroidaceae*, and *Lachnospiraceae*

To determine (1) which OTUs varied in abundance between groups and (2) the contribution of distinct OTUs to the variance between groups, we performed a paired *T* test with a Benjamin-Hochberg correction for false discovery (http://www.biostathandbook.com/multiplecomparisons.html) and similarity percentages analysis. In summary, ^Hu^microbiota and ^Conv^microbiota samples could be distinguished by OTUs assigned to the *Bacteroidetes* and *Firmicutes* phyla, and these phyla contributed to 57.19 and 26.6% of the variance between TSB sham-inoculated ^Hu^microbiota and ^Conv^microbiota mice, respectively (Table 3). The most abundant OTUs contributing to the difference between the groups are OTUs 002, the dominant OTU in the ^Conv^microbiota samples, and OTUs 001 and 003, which are the dominant OTUs in the ^Hu^microbiota samples. Collectively, OTUs 001, 002, and 003 contribute 57.93% of the variance between TSB sham-inoculated ^Hu^microbiota and ^Conv^microbiota mice.

**Table 3:** Contribution of taxa to group differences. The average read abundance of OTUs that distinguish the Hu-TSB and Conv-TSB based *P* values were determined by paired *T* test with Benjamin-Hochberg correction for multiple comparisons. Contribution to variance was determined by Similarity Percentages (SIMPER) analysis

	Average abundance								
OTU	Conv-TSB	Hu-TSB	*P* value	% Contribution	Phylum	Class	Order	Family	Genus
002	2070.3	32.9	≤0.0001	22.73	*Bacteroidetes*	*Bacteroidia*	*Bacteroidales*	*Porphyromonadaceae*	unclassified
003	0.5	1641.2	≤0.0001	23.18	*Bacteroidetes*	*Bacteroidia*	*Bacteroidales*	*Bacteroidaceae*	*Bacteroides*
004	121.5	76.4	≤0.0651	0.58	*Bacteroidetes*	unclassified	unclassified	unclassified	unclassified
006	47.7	515.2	≤0.0001	6.04	*Bacteroidetes*	*Bacteroidia*	*Bacteroidales*	unclassified	unclassified
008	0.6	363.6	≤0.0001	4.28	*Bacteroidetes*	*Bacteroidia*	*Bacteroidales*	*Porphyromonadaceae*	*Parabacteroides*
014	14.4	0.4	≤0.0004	0.38	*Bacteroidetes*	*Bacteroidia*	*Bacteroidales*	*Porphyromonadaceae*	*Barnesiella*
001	522.6	1111.1	0.0619	12.02	*Firmicutes*	*Clostridia*	*Clostridiales*	Lachnospiraceae	unclassified
005	231.1	69.3	0.0003	2.77	*Firmicutes*	*Clostridia*	*Clostridiales*	unclassified	unclassified
009	73.5	102.5	0.1230	0.75	*Firmicutes*	*Clostridia*	*Clostridiales*	*Ruminococcaceae*	unclassified
010	88	5.5	≤0.0001	1.07	*Firmicutes*	unclassified	unclassified	unclassified	unclassified
011	521.6	167.6	0.0146	5.20	*Firmicutes*	*Erysipelotrichia*	*Erysipelotrichales*	*Erysipelotrichaceae*	*Turicibacter*
012	297.2	0.1	≤0.0001	2.58	*Firmicutes*	*Bacilli*	*Lactobacillales*	*Lactobacillaceae*	*Lactobacillus*
016	7.6	97.8	0.0010	0.99	*Firmicutes*	*Clostridia*	*Clostridiales*	*Lactobacillaceae*	*Clostridium*_XIVa
028	3.7	1.9	0.0453	0.04	*Firmicutes*	*Clostridia*	*Clostridiales*	*Lactobacillaceae*	*Dorea*
032	35.8	0.6	0.0019	0.80	*Firmicutes*	*Clostridia*	unclassified	unclassified	unclassified
056	1.6	14.5	0.0004	0.17	*Firmicutes*	*Clostridia*	*Clostridiales*	*Ruminococcaceae*	*Flavonifractor*
060	0.8	0.1	0.0352	0.03	*Firmicutes*	*Clostridia*	*Clostridiales*	Lachnospiraceae	*Johnsonella*
025	0.1	12	0.0019	0.23	*Proteobacteria*	*Betaproteobacteria*	unclassified	unclassified	unclassified
039	62.5	0.1	0.0223	1.20	*Tenericutes*	*Mollicutes*	*Anaeroplasmatales*	*Anaeroplasmataceae*	*Anaeroplasma*
007	206.2	25.5	≤0.0001	2.77	unclassified	unclassified	unclassified	unclassified	unclassified

Eigenvalues and loadings for the principal components analysis shown in Fig. 4 are given in Additional file 2 Table S1 and are similar to the results of the similarity percentages analysis. Both inspection of the scree plot (not shown) and application of the Joliffe cutoff to the eigenvalues indicate that the first four axes are the most significant axes. In the PCA, the eigenvalues of the first two axes together account for 89.6% of the variance; loadings indicate that the first axis of the PCA, which separates humanized from conventional microbiota mice, is dominated by OTUs 2 and 3 (unclassified member of the family *Prophyromonadaceae* and a member of the genus *Bacteroides*, respectively). Axis 2 is dominated by OTUs 1 and 3 (unclassified member of the family *Lachnospiraceae* and a member of the genus *Bacteroides*, respectively).

Additional file 3: Table S2 lists a relatively small number of OTUs identified by mothur as significantly associated with either humanized or conventional microbiota mice (i.e., indicator OTUs). Because the genus *Lactobacillus* appears in the conventional microbiota list, it is tempting to speculate that the organism represented by this OTU is protective against *Campylobacter* colonization or modulates immune responses in conventional microbiota mice or both. However, it is important to note that not all species, or even all strains within a species, of the genus *Lactobacillus* have probiotic effects. It is equally possible that some process carried out by one or more of the organisms represented by the OTUs in the humanized microbiota mice list enables *Campylobacter* colonization or modulates immune responses toward Th2 pathways in humanized microbiota mice.

### Disease indicators: ^Hu^microbiota mice displayed increased susceptibility to intestinal inflammation

**Fig. 6:**
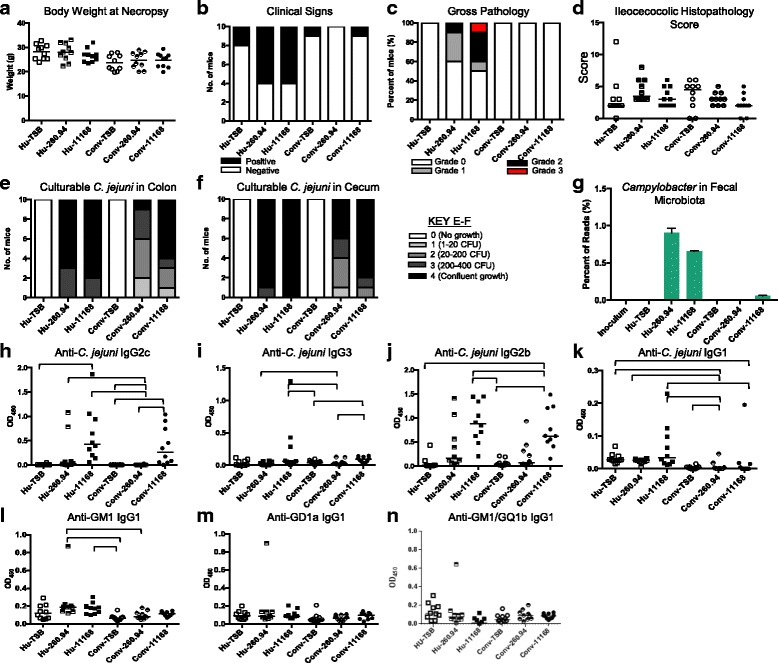
^Hu^microbiota mice are more susceptible to *C. jejuni* colonization, GI inflammation and antiganglioside antibodies than ^Conv^microbiota mice. Data represent (**a**) body weight at necropsy, (**b**) clinical signs, (**c**) gross pathology, (**d**) ileocecocolic histopathology scores, (**e**) culturable *C. jejuni* in colon, (**f**) culturable *C. jejuni* in cecum, and (**g**) percentage of 16S rRNA amplicons assigned to the genus *Campylobacter*. Panels **h** to **k** are isotype specific anti-*Campylobacter* antibody responses in plasma detected by indirect ELISA. Panels **l**, **m** and **n** show anti-ganglioside antibody responses in plasma detected by indirect ELISA. Data were analyzed by Kruskal-Wallis test on ranks and Dunn’s post-test where appropriate; p≤0.05 considered statistically significant

To compare the progression and severity of disease in experimental mice, we monitored all mice for (1) clinical signs, (2) gross pathology, and (3) disparity in body weight. All mice were monitored closely by veterinarians to determine if euthanasia was required prior to the scheduled 7-week endpoint. These determinations were based on hunching, lethargy, and watery or bloody diarrhea in accordance with a standardized scoring system [30] available from the Michigan State University Microbiology Research Unit Food and Waterborne Diseases Integrated Research Network-sponsored Animal Model Phenome Database for gastrointestinal disease and another score sheet developed to monitor development of neurological disease that might have developed in mice given the GBS-associated *C. jejuni* strain 260.94 [33]. No severe disease indicators were detected during the experimental course that exceeded the scoring limit of 9 for humane euthanasia, thus all of the mice were maintained for the entirety of the experiment. No significant differences in body weight were detected, although some ^Hu^microbiota mice were heavier than ^Conv^microbiota mice in all groups (Fig. 6a). Clinical signs were detected mainly in two groups, those that were ^Hu^microbiota mice infected with either *C. jejuni* 260.94 or 11168 (Fig. 6b). Six of ten ^Hu^microbiota mice infected with *C. jejuni* 260.94 had episodes of soft feces, hunched posture, rough hair coat, and reduced activity over the 7-week period; six of ten ^Hu^microbiota mice infected with *C. jejuni* 11168 also had episodes of soft feces, hunched posture, rough hair coat, and reduced activity over the 7-week period. During this period, only one of ten ^Conv^microbiota mice given *C. jejuni* 260.94 or 11168 had soft feces and no other clinical signs. Control mice had virtually no clinical signs except for one sham-inoculated ^Conv^microbiota mouse judged to have reduced activity on one occasion and two sham-inoculated ^Hu^microbiota mice judged to have a rough hair coat on one occasion. Four of ten *C. jejuni* 260.94 infected and five of ten *C. jejuni* 11168 infected ^Hu^microbiota mice had severe gross pathological changes in the GI tract (Fig. 6c). In many of these cases, the ileocecocolic lymph node, spleen, and sometimes the mesenteric lymph nodes were enlarged. One *C. jejuni* 11168 infected ^Hu^microbiota mouse had a slightly thickened colon wall. To determine if the level of *C. jejuni* differed between ^Hu^microbiota and ^Conv^microbiota mice, a potential cause of enhanced GI gross pathology, we quantified *C. jejuni* in both the cecum and colon. Colonization was significantly higher in ^Hu^microbiota than ^Conv^microbiota mice (Fig. 6e, f). These data were supported by a 10- and 100-fold increase in *Campylobacter* reads in ^Hu^microbiota fecal microbiota analysis of mice infected with *C. jejuni* strains 11168 and 260.94, respectively, compared to ^Conv^microbiota fecal microbiota samples (Fig. 6g). We processed the ileocecocolic junctions for histopathologic evaluation of the ileum, cecum, and colon and found that although a few C57BL/6 ^Hu^microbiota mice had higher scores than their congenic ^Conv^microbiota counterparts, gastrointestinal lesions were mild and not significantly different between groups. Thus, the main changes associated with experimental *C. jejuni* infections were in secondary lymphoid tissues including the ileocecocolic lymph nodes, the mesenteric lymph nodes, and the spleen in ^Hu^microbiota mice.

### Mice with ^Hu^microbiota had greater T_H_2-dependent IgG1 responses to both enteric and GBS strains of *C. jejuni*

To assess specific immune reactivity and cross-reactive antibody responses, we measured anti-*C. jejuni* and anti-ganglioside antibody responses in infected mice by indirect ELISA. Both ^Hu^microbiota and ^Conv^microbiota mice mounted anti-*C. jejuni* IgG2c (T_H_1 associated) and IgG2b (T_H_17 associated) antibody responses to 11168 when compared to their respective TSB sham-inoculated controls (Fig. 6h, j). However, the anti-*C. jejuni* IgG2b responses to strain 11168 in ^Hu^microbiota mice were significantly higher than in ^Conv^microbiota mice, while the anti-*C. jejuni* IgG2c responses were not significantly different between these groups (Fig. 6h, j). This may simply indicate that anti-*C. jejuni* T helper 1 (T_H_1) responses wane faster than T_H_17 responses in this model at the 7-week time point. Yet the anti-*C. jejuni* IgG2c (T_H_1 associated) and IgG2b (T_H_17 associated) antibody responses to 260.94 were higher in this experiment, with ^Hu^microbiota mice producing significantly higher anti-*C. jejuni* IgG2c responses than ^Conv^microbiota mice (Fig. 6h, j). Furthermore, ^Hu^microbiota mice produced significantly higher anti-*C. jejuni* IgG1 responses than ^Conv^microbiota mice to both *C. jejuni* strains (Fig. 6k). In TSB sham-inoculated mice, T_H_2-associated anti-*Campylobacter* IgG1 antibody responses were elevated in ^Hu^microbiota mice compared to ^Conv^microbiota mice, indicating a T_H_2-associated antibody bias in ^Hu^microbiota mice that was not associated with the presence of the inoculating bacterium (Fig. 6k). This was consistent with results in the pilot experiment. Finally, the anti-*C. jejuni* IgG3 responses to strain 11168 in ^Hu^microbiota mice were significantly higher than those seen in ^Conv^microbiota mice given TSB or *C. jejuni* 260.94 (Fig. 6i). Significant elevations in anti-*C. jejuni* IgG3 responses were seen in ^Conv^microbiota mice given *C. jejuni* 11168 compared to those given TSB or *C. jejuni* 260.94 but these responses were minimal (Fig. 6i).

### Mice with ^Hu^microbiota had greater anti-GM1 ganglioside antibody responses than mice with ^Conv^microbiota

Based on the pilot experiment and because IgG1-specific anti-ganglioside responses have been associated with development of GBS in human patients, we measured anti-GM1 and GD1a single ganglioside responses in Experiment 1. Similarly to the pilot experiment results, we found that ^Hu^microbiota mice infected with *C. jejuni* 260.94 had significantly elevated anti-GM1 IgG1 antibodies when compared to ^Conv^microbiota mice infected with the same strain (Fig. 6l). However, unlike results in the pilot experiment, ^Hu^microbiota mice infected with *C. jejuni* 11168 had significantly elevated anti-GM1 IgG1 antibodies when compared to the sham-inoculated ^Conv^microbiota mice, but not when compared to the ^Conv^microbiota mice infected with the same strain (Fig. 6l). This difference correlated with slightly lower anti-*Campylobacter* and anti-ganglioside antibodies at 7 weeks post infection versus 5 weeks post infection in the pilot experiment (Figs. 1g–m and 6h–n). No significant anti-GD1a or anti-GM1/GQ1b responses were seen.

### *C. jejuni* 11168-infected ^Hu^microbiota mice had significantly higher IL-4 colon responses at 5 but not 7 weeks post infection

**Fig. 7:**
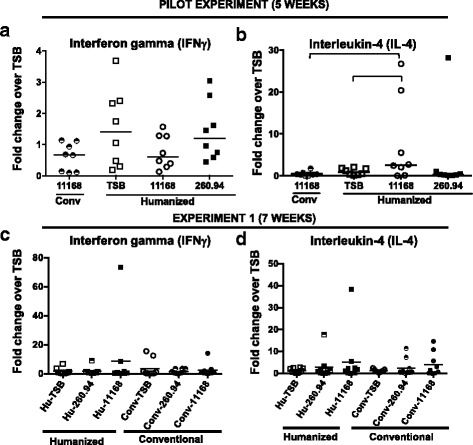
*C. jejuni* infected ^Hu^microbiota mice have significantly elevated colon IL-4 cytokine responses at five but not seven weeks post infection when compared to sham inoculated ^Hu^microbiota mice or *C. jejuni* infected ^Conv^microbiota mice. Panels represent fold change in interferon gamma (IFN-γ) (**a** and **c**) and IL-4 responses (**b** and **d**) over responses of the sham inoculated mice. Panels **a** and **b** are from mice in the Pilot experiment sacrificed at 5 weeks post infection while panels **c** and **d** are from mice in Experiment 1 sacrificed at 7 weeks post infection. IFN-γ and IL-4 mRNA levels were measured in proximal colon homogenates from all mice in the respective groups shown on the X axis. Data were analyzed by Kruskal-Wallis test on ranks and Dunn’s post-test where appropriate; p≤0.05 was considered statistically significant

After conducting both the pilot experiment and Experiment 1, we processed snap frozen colon tissues for measurement of T_H_1-associated inflammatory (IFNγ) and T_H_2-associated anti-inflammatory (IL-4) mRNA levels using RT-PCR. At 5 weeks post infection (PI) in the pilot experiment, *C. jejuni* 11168-infected ^Hu^microbiota mice had significantly higher IL-4 colon responses when compared to TSB sham-inoculated controls and to ^Conv^microbiota mice infected with the same strain (Fig. 7b). No other significant differences in cytokine measurements were seen at either 5 or 7 weeks post infection in either experiment (Fig. 7a–d). In Experiment 1, three high reactor mice were detected for IL-4 responses in the following groups, Hu-260.94 (19.4-fold increase/HPRT compared to group mean of 1.7), Hu-260.94 (17.8-fold increase/HPRT compared to group mean of 1.7), and Hu 11168 (38.3-fold increase/HPRT compared to group mean of 5.244) (Fig. 7d). There was a single high reactor for IFNγ responses in ^Hu^microbiota mice given *C. jejuni* 11168 (Fig. 7c). These high reactors correlated with *C. jejuni* infection status and the otherwise low responses at 7 weeks PI likely reflect waning of early colon responses. Subsequent analyses were conducted to determine if *C. jejuni* infection initiated autoimmune responses to peripheral nerves consistent with the predicted mechanism of GBS. To determine if macrophage numbers in sciatic nerves and dorsal root ganglia were increased in *C. jejuni-*infected mice compared to controls, we immunohistochemically labeled these tissues with anti-F4/80 antibody and counted positive cells. No differences in peripheral nerve lesions were detected.

### ^Hu^microbiota mice display hypoactivity in the open-field test and infection alters activity in mice with ^Conv^microbiota

**Fig. 8:**
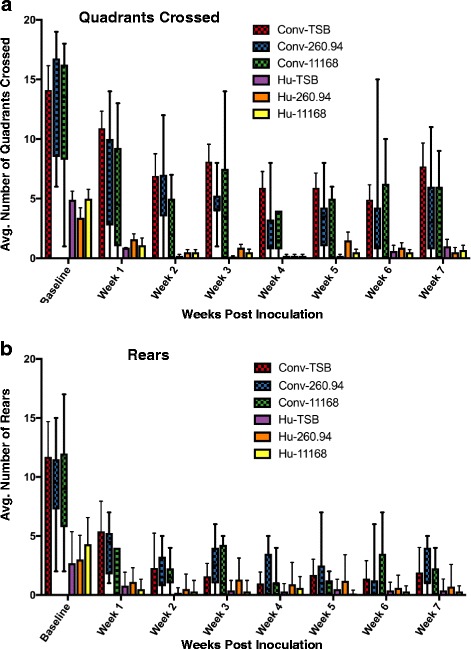
Behavioral phenotyping in the open-field test. Number of (**a**) Quadrants crossed and (**b**) Rears in the open-field one-week prior to inoculation (i.e. baseline) and 1 to 7 weeks post-inoculation. Boxes represent 95% confidence intervals and whiskers represent range. Lines represent the median of ^Conv^microbiota (red) and ^Hu^microb﻿iota (blue) mice regardless of inoculation status. Data were analyzed by repeated measures two-way analysis of variance (ANOVA) and Tukey’s post-test; p≤0.05 indicates statistical significance (reported in results).﻿ The key shows the color of each experimental group

To determine if *C. jejuni* infection was associated with behavioral phenotypes indicative of enteric disease or peripheral neuropathy, we recorded the activity of the experimental mice in the open-field test. In general, mice with ^Hu^microbiota displayed diminished activity levels compared to ^Conv^microbiota mice regardless of infection status (Fig. 8a, b), and both groups trended toward a decrease in activity as time progressed. Significant decreases in the number of quadrants crossed were primarily dependent upon having ^Hu^microbiota; no differences were detected within the ^Hu^microbiota mice during the 7 weeks, and they generally displayed low levels of activity. After week 3, no differences in rearing behavior were detected between any groups as mice were generally inactive.

## Discussion

*C. jejuni* is a leading cause of bacterial diarrheal illness and the leading antecedent infection to the autoimmune acute peripheral neuropathy GBS [21, 34]. In previous work, we developed mouse models of both enteric and subsequent neurologic disease associated with *C. jejuni* infection [30, 31, 33, 35]. In the experiments reported here, we explored the influence of the gut microbiota on these disease manifestations. After six generations of breeding, individually housed C57BL/6 mice with a transplanted human microbiota were infected with *C. jejuni* enteric disease or GBS patient strains. These mice retained a microbiota distinct from that of their ^Conv^microbiota counterparts that could be primarily distinguished by bacteria belonging to the phyla *Bacteroidetes* and *Firmicutes. C. jejuni* infection did not appear to alter the microbiome composition in either group (Fig. 4b, c). Moreover, the abundance of *Lactobacillus*, which has been shown to prevent *C. jejuni* colonization in in vivo and in vitro models [26, 36], was at least 6000-fold greater in ^Conv^microbiota than ^Hu^microbiota mice. Consistent with those results [26, 36], semi-quantitative and 16S rRNA gene amplicon analysis showed that microbiota source affected *C. jejuni* load. Ten-fold higher *C. jejuni* 11168 and 100-fold higher *C. jejuni* 260.94 loads were detected in the fecal microbiota of infected ^Hu^microbiota mice than their ^Conv^microbiota-matched counterparts (Fig. 6g). Furthermore, the presence of this ^Hu^microbiota skewed adaptive T cell responses to *C. jejuni*. Mice with ^Hu^microbiota had greater T_H_2-dependent IgG1 responses to both enteric and GBS strains of *C. jejuni* and greater anti-GM1 ganglioside antibody responses than mice with ^Conv^microbiota. These results confirm that this T cell skewing favored development of autoimmune antibodies directed against nerve gangliosides, which is a hallmark of a GBS response. Additionally, we observed that this ^Hu^microbiota caused mixed T_H_1/T_H_17/T_H_2-associated responses to occur to a strain of *C. jejuni* 260.94 that was previously shown to produce only T_H_2 responses in C57BL/6 IL-10^−/−^ mice with ^Conv^microbiota [31]. The shift in T cell responsiveness in the experiments reported here was accompanied by the occurrence of distinct disease manifestations in the GI tract and the presence of anti-ganglioside antibodies in infected ^Hu^microbiota mice as compared to the infected or uninfected ^Conv^microbiota mice. *C. jejuni* 11168 or 260.94 infected ^Hu^microbiota mice had increased gross gastrointestinal lesions, which was not seen previously in ^Conv^microbiota mice of the same genotype from the same breeding colony [31]. Overall, our results support the conclusion that components of the murine microbiota play a role in colonization resistance that is overcome in our ^Hu^microbiota model, resulting in increased susceptibility to both inflammation and autoimmunity.

We analyzed *C. jejuni*-infected mice with ^Hu^microbiota and ^Conv^microbiota for enteric inflammation and found that ^Hu^microbiota mice are more susceptible to *C. jejuni-*mediated inflammation determined by having more severe gross pathology especially enlarged lymph nodes. These results are consistent with previous reports showing that the microbiota is a key regulator of enteric disease in *C. jejuni*-infected mice [24–26]. Despite this finding, significant enteric histologic lesions were not found and inflammatory cytokine levels were not significantly elevated in the proximal colon of ^Hu^microbiota mice. These results suggest that most of the immune reactivity was occurring in the draining lymph nodes and not in the colon wall in most infected mice. We previously reported that interleukin-10 deficiency significantly increased host-inflammatory responses to *C. jejuni*. Now these results indicate that ecological shifts in the microbiota are another factor sufficient to enhance host susceptibility to *C. jejuni* resulting in mild enteric disease in IL-10-sufficient mice.

Infection with *C. jejuni* GBS patient strains is associated with T_H_2 immune responses and anti-ganglioside antibodies in a C57BL/6 IL-10-deficient mouse model [31]. In these experiments, ^Hu^microbiota mice showed a type 2-biased antibody response with or without infection compared to ^Conv^microbiota mice. Analysis of anti-ganglioside antibodies showed exacerbated anti-GM1 antibody levels following infection with *C. jejuni* 260.94 but not enteric strain 11168 compared to ^Conv^microbiota mice infected with the same *C. jejuni* strain. We have demonstrated microbiota-mediated autoimmunity in *C. jejuni*-infected mice with depleted microbiota previously (Brooks et al. unpublished); however, this is the first study to show that ecological shifts in a diverse microbial community are sufficient to alter *C. jejuni*-mediated autoimmunity. In fact, while T_H_1/T_H_17 responses (IgG2c, IgG2b) to the *C. jejuni* 11168 strain in ^Hu^microbiota mice are similar to those in IL-10-deficient mice, ^Hu^microbiota mice have more pronounced T_H_2 responses to *C. jejuni* 11168 than IL-10-deficient mice and yet displayed no demonstrable nerve lesions of GBS. This result suggests that anti-ganglioside antibodies alone cannot produce GBS in the ^Hu^microbiota C57BL/6 mice where IL-10 is present. These results, in conjunction with anti-*Campylobacter*-specific antibody results show that differences in the lipooligosaccharide outer core antigens presented on the two *C. jejuni* strains are not the only factor driving enteric and GBS phenotypes. In addition, presence of *C. jejuni* 11168 IgG1 antibody responses also suggest that the 11168 strain is not strictly an enteritis-causing strain. Although it was taken from a human patient with acute gastroenteritis, the possibility that a single strain could cause different manifestations of disease in different individuals, perhaps even depending on the microbiota composition of those individuals, should not be discounted.

A significant decrease in activity in the open-field test was detected in all ^Hu^microbiota mice, while overt neurological signs of GBS were not significantly elevated in the infected versus TSB sham-inoculated groups. The open-field test has been used as a non-invasive longitudinal measure of quality of life that allows the investigator to make inferences about anxiety and normal exploratory behavior [37–39]. It also can be used to assess ambulation and rearing that when quantified with the JWatcher software can provide indication of motor and proprioception impairment. Our mice were observed by veterinarians trained to assess neurological behavior in animals. However, this decrease in activity was not due to infection status, nor were there any severe clinical signs of neurologic or enteric disease detected in the ^Hu^microbiota mice; thus we conclude that this decreased activity is not an indicator of nervous system disease in response to infection. Moreover, sciatic nerves and lumbar dorsal root ganglia were dissected and immunohistochemically labeled and macrophages quantified to determine whether an increase in macrophages could be detected as a potential mechanism of peripheral nerve damage consistent with that seen in patients with GBS [12, 21, 40]. An increase in macrophages in or around the nerve is indicative of complement-dependent injury; however, no differences in macrophage numbers were detected in our study. Furthermore, mere changes in weight of the mice were not great enough to explain this inactivity. Thus, our results suggest the possibility of an influence of the microbiota on brain and behavior, which has been shown previously [41–43]. Taken together, these findings suggested that the particular microbiota influenced stress responses in the ^Hu^microbiota mice independent of the infection status. Further investigation is required to determine the origin of this response in the ^Hu^microbiota mice.

In summary, we found that the microbiota is a key factor in the regulation of *Campylobacter* inflammation in the intestine and the elicitation of anti-ganglioside antibodies. These data support recent findings that the microbiota is a critical component in *Campylobacter* gastroenteritis and, to our knowledge, this is the first report to suggest that the microbiota may in fact be a determinant of host susceptibility to Guillain-Barré syndrome. *C. jejuni* microbiota-mediated colonization resistance in mice is overcome by perturbation of the microbiota; thus factors that alter the host microbiota such as age, diet, antibiotic treatment, and prior pathogen exposure may be determinants of susceptibility to Guillain-Barré syndrome. Because no single human microbiota exists, it is reasonable to speculate that OTUs distinguishing the human and murine microbiota in our experimental mice play a role in regulating *C. jejuni* loads. Finally, therapeutic approaches that avoid depletion of healthy microbiota and enhance populations of beneficial microorganisms may be appropriate. Recently, probiotics, including *Lactobacillus*, have been shown to inhibit *C. jejuni* growth in mice [26]; thus probiotic supplementation during the early stages of infection may facilitate clearance of *C. jejuni* [44].

## Conclusions

These data demonstrate that ^Hu^microbiota altered host-pathogen interactions in infected mice, increasing colonization and Th-2 and autoimmune responses in a *C. jejuni* strain-dependent manner. This demonstrates that colonic microbiota composition is another factor controlling susceptibility to GBS. This study and the resulting animal model provides the basis for understanding how these autoimmune neurological responses arise secondary to an important foodborne pathogen.

## Methods

### Laboratory animals

C57BL/6J and C56BL/6 IL-10^−/−^ mice were obtained from The Jackson Laboratories (Bar Harbor, ME). A breeding colony was established in a *Campylobacter*/*Helicobacter*-free facility, and the MouSeek database (Caleb Davis, Baylor College of Medicine, Houston, TX) was used to track all mice bred and used throughout the study. Germ-free C57BL/6J mice were also obtained from the same containment building at The Jackson Laboratories. All mouse experiments were performed according to recommendations in the Guide for the Care and Use of Laboratory Animals of the National Institutes of Health. Protocols were reviewed and approved by the Michigan State University Institutional Animal Use and Care Committee (approval numbers 06/12-107-00 and 06/15-101-00). Age-matched male and female mice were used for all experiments. A portion of the mice in each experiment possessed humanized microbiota generated as described previously [45] and are indicated with the prefix Hu (^Hu^microbiota). Briefly, germ-free mice were inoculated by gavage with a human fecal slurry, bred, and the microbiota allowed to pass from mother to offspring without intervention within the germ-free incubator. After initial characterization in founder mice described in Collins et al. 2015 [45], the ^Hu^microbiota mice were separated into two groups, and a new colony was established (Hu-C57BL/6) by LS Mansfield by transferring mice in sterile filter top cages within sterile dog crates to Michigan State University. ^Hu^microbiota mice were housed under specific pathogen-free conditions (SPF), and bred for six generations in closed cages on an Innovive (San Diego, CA, USA) mouse rack with filtered air flow and sterile food and water. All cage changes and other manipulations were performed in a laminar flow hood with gowned and gloved personnel using sterile technique to avoid introduction of microorganisms from the environment.

In the pilot experiment, age-matched 10–12-week-old ^Hu^microbiota C57BL/6 genetically wild-type (Hu), conventional microbiota genetically wild-type (^Conv^microbiota), and ^Conv^microbiota congenic IL-10-deficient mice (Conv-IL-10^−/−^) were used. Mice were inoculated with either tryptone soy broth (TSB; vehicle control), *C. jejuni* 260.94, or *C. jejuni* 11168 and handled with sterile or specific pathogen-free (SPF) technique resulting in six groups (Table 1). For SPF technique, all personnel that were handling animals wore Tyvek coveralls, impermeable plastic booties, face mask, hair bonnet, and gloves. All cage changes were performed on a laminar flow cage changing station. For sterile technique, all personnel that were handling animals wore impermeable plastic booties, face mask, hair bonnet, sterile surgical gown, and sterile surgical gloves. All breeding mice were and continue to be handled using sterile technique to avoid introducing extraneous organisms to the microbiota. All cage changes for breeding mice were performed in a sterile laminar flow hood that was disinfected after each use. To determine if handing would alter outcomes in TSB sham-inoculated ^Hu^microbiota mice, we inoculated 20 ^Hu^microbiota mice with TSB and handled them with either sterile technique (Hu-TSB (Ster.)) or SPF technique (Hu-TSB (SPF)). Two other ^Hu^microbiota groups were generated by inoculating ^Hu^microbiota mice with either *C. jejuni* 260.94 (Hu-260.94 (Ster.)) or *C. jejuni* 11168 (Hu-11168 (Ster.)) and handling them with sterile technique. As a positive control for gastroenteritis, we inoculated and compared outcomes in ^Conv^microbiota wild-type C57BL/6 and C57BL/6 IL-10^−/−^ mice inoculated with *C. jejuni* 11168 and handled with SPF technique. Mice were sacrificed at 5 weeks post-inoculation.

In Experiment 1, age-matched C57BL/6 ^Hu^microbiota and ^Conv^microbiota mice were inoculated with TSB, *C. jejuni* 260.94, or *C. jejuni* 11168 (Table 2). In Experiment 1, all mice were handled with SPF technique, observed for 7 weeks post-inoculation, and then sacrificed. In all, six experimental groups were generated in Experiment 1; ^Conv^microbiota TSB inoculated (Conv-TSB), ^Conv^microbiota *C. jejuni* 260.94 infected (Conv-260.94), ^Conv^microbiota *C. jejuni* 11168 infected (Conv-11168), ^Hu^microbiota TSB inoculated (Hu-TSB), ^Hu^microbiota 260.94 infected (Hu-260.94), and ^Hu^microbiota 11168 infected (Hu-11168).

### Enteric pathogen screening

DNA was extracted from feces collected from all mice before experimental inoculation and at necropsy for enteric pathogen screening as described [30]. In all cases, no control mice were positive for *C. jejuni* PCR using gyrA-specific primers [46]. Also, we screened all samples for *Campylobacter* spp. (16S rRNA gene), *Helicobacter* spp. (16S rRNA gene), *Citrobacter rodentium* (*esp*B gene), and *Enterococcus faecalis* (*ddl* gene). Dedicated sentinel mice were used to assess extraneous infection with bacteria, protozoa, and viral agents (Charles River Laboratories, Wilmington, MA) and were monitored by the MSU Campus Animal Resources (CAR).

### *C. jejuni* strains and inoculum preparation

*C. jejuni* strains 260.94 (ATCC BAA-1234) and NCTC 11168 (ATCC 700819) were obtained from the American Type Culture Collection (Manassas, VA). *C. jejuni* 260.94 is a Guillain-Barré syndrome patient strain that elicits GM1 and GD1a anti-ganglioside antibody responses in C57BL/6 IL-10^−/−^ mice [31]. *C. jejuni* 11168 is an enteric disease patient strain isolated from a patient with severe gastroenteritis. *C. jejuni* 11168 has a GM1 ganglioside mimic on its surface [47] but is not associated with GBS and has not been shown to elicit significant anti-ganglioside antibody responses in C57BL/6 IL-10^−/−^ mice [31]. Inocula were prepared in the same manner for both experiments. Inocula of both *C. jejuni* strains were prepared by streaking frozen stocks onto tryptone soy agar (TSA) (Accumedia) supplemented with 5% defibrinated sheep blood (Cleveland Scientific, Bath Ohio) (TSAB). Plates were incubated at 37 °C in anaerobic jars equilibrated to 10% CO_2_, 10% H_2_, and 80% N_2_ for 48 h and a portion of the growth re-suspended in tryptone soya broth (TSB) to give an A600 of 0.2 to 0.3. One-hundred microliters of this suspension was spread on two plates per mouse and the plates incubated for 16 h in the 10% CO_2_, 10% H_2_, and 80% N_2_ gas mixture. The resulting cells were collected and suspended in TSB; the suspension was adjusted to give an A600 of approximately 1.0 when diluted 1:10 (approximately 1 × 10^10^ CFU/mL final concentration). Purity, morphology, and motility were verified by microscopy and Gram straining. Finally, 0.2 mL per mouse of the resulting inoculum or the vehicle (i.e., TSB) was carried to the containment facility on ice and delivered to infected and control mice, respectively, by oral gavage, resulting in six groups (Tables 1 and 2). Limiting dilution analysis was used to determine the actual titer of the inoculum delivered to the mice.

### Experimental design

Following infection, all mice were observed at least once daily (twice daily after clinical signs were noted) by trained individuals for a period of 5 (Pilot) or 7 (Experiment 1) weeks to ensure mice were euthanized at a humane endpoint. In Experiment 1, 1 week before infection (i.e., baseline) and once each week for 7 weeks post-inoculation, mice underwent behavioral phenotyping in an open-field test in a sterile rat cage (18′′ × 8′′) divided into four quadrants located in a laminar flow hood. At 5- (Pilot) or 7-weeks (Experiment 1), the mice were sacrificed, and tissues were collected and stored for further analysis. Prior to humane euthanasia by CO_2_ overdose, fecal samples were collected, placed in TSB, frozen on dry ice, and quickly moved to a −80 °C freezer until thawing for DNA extraction. After euthanasia, mice were weighed and blood was collected by cardiac puncture, immediately mixed with 0.1 mL of 3.8% citrate, spun down, and plasma stored at −80 °C for analysis of plasma antibodies. During necropsy, two veterinarians (a pathologist and a gastroenterologist) observed and recorded any gross pathology prior to the removal of the GI tract. For the Pilot and Experiment 1, the cecum and colon were harvested, cut in half, and the halves flash frozen or streaked on TSAB-CVA plates for cytokine analysis and quantification of *C. jejuni* in these compartments, respectively. In Experiment 1, the ileocecocolic junction was harvested, infiltrated with 10% neutral-buffered formalin (NBF), placed in a cassette, and further fixed in NBF for 20–24 h and stored in 60% ethanol until processed for histological analysis.

### Bacterial DNA isolation from feces and 16S ribosomal RNA gene analysis

In the pilot experiment, DNA was extracted from fecal samples using the QIAamp DNA stool kit (QIAGEN) according to manufacturer’s instructions. DNA concentrations were determined using a NanoDrop ND-1000 spectrophotometer and concentrations normalized. The quantity of *Clostridium* group 1, *Clostridium* group 1, *Bacteroidetes*, and *Enterobacteriaceae* were measured using an IQ^TM^5 Multicolor Real-Time PCR Detection System. In Experiment 1, DNA was extracted from fecal samples using bead beating and the FastDNA SPIN Kit for Soil (MP Biomedicals, LLC) according to manufacturer’s instructions. The resulting DNA samples were delivered to the Michigan State University Research Technology Support Facility for library preparation and 16S rRNA gene amplicon analysis. In all, 62 samples were submitted for sequencing, including 60 mouse samples, the original fecal slurry used for inoculation of founder mice, and a mock community (HM-782D, BEI) for estimation of sequencing error. The V4 region of the 16S rRNA gene was amplified using dual-indexed primers [48]. PCR products were normalized using an Invitrogen SequalPrep DNA Normalization plate and the normalized products pooled. After quality control and quantitation, the pool was loaded on a standard MiSeq v2 flow cell and sequenced with a 500 cycle MiSeq v2 reagent kit (paired-end 250 base pair reads). Base calling was performed by Illumina Real-Time Analysis (RTA) v1.18.54 and output of RTA was de-multiplexed and converted to FastQ format files with Illumina Bcl2fastq v1.8.4.

16S rRNA gene amplicon analysis was performed using mothur (v. 1.35) and protocols available at http://www.mothur.org/wiki/MiSeq_SOP accessed December, 2015. Alignment was achieved using the Silva 16S ribosomal gene database [49]. Chimeric sequences and any sequences classified as chloroplast, mitochondria, Archaea, or Eukaryota were removed from the dataset using uchime and the mothur formatted version of the Ribosomal Database Project (RDP) training set version 9, respectively, per the mothur protocol. Sequences were clustered in operational taxonomic units (OTUs) of 97% sequence identity yielding 128 OTUs. Analyses were performed in mothur and PAST 3.07 [50]. Sequence read data has been made available in the National Center for Biotechnology Information (NCBI) Sequence Read Archive (SRA) as documented in “Availability of data and materials.” A full record of the code used to develop the heat map that appears in Fig. 2, is based on the mothur protocol cited above. An annotated markdown file with the code for the heat map appears in Additional files 4 and 5.

### Clinical signs assessments

We used a clinical sign score sheet developed to discern humane endpoints for gastrointestinal and neurological disease in mice; these have been approved by the MSU institutional animal care and use committee (IACUC) and published [30, 33, 35]. Briefly, mice were observed once a day by trained animal handlers and when clinical signs were discerned, they were documented and the mice were thereafter observed twice a day. A score sheet was filled out each time a mouse showed a clinical sign. Each sign has a point value and the scores for all signs observed were totaled for that observation period. If the score equaled or exceeded 9 then the mouse was humanely euthanized. Mice were assigned scores for a battery of clinical signs according to this scoring system: (1) Eating/Drinking (0 = yes, 1 = No); (2) Respiration (0 = normal, 1 = abnormal (increased), 10 = labored); (3) Rough hair coat (0 = no, 2 = yes), Hunched posture (0 = no, 9 = yes), Tremors (0 = no, 10 = yes), Movement (0 = normal, 1 = subdued (moves with stimulation), 2 = unresponsive to handling), Crusty eyes (0 = no, 1 = one eye, 2 = 2 eyes), Diarrhea on fur (0 = no, 1 = yes), Cool to the touch (0 = no, 10 = yes), and Body weight (0 = 0–1% weight loss, 1 = 1–5% weight loss). Endpoints resulting in a score greater than 9 include loss of body weight greater than 5%, cool to touch, blue extremities, or points adding up to greater than 9 in other criteria.

### Quantification of *C. jejuni* in the cecum and colon

*C. jejuni* in the colon and cecum were quantified using a standardized semi-quantitative scoring system [30]. Briefly, colon and cecum tissue segments of the same size were collected at necropsy and were streaked on TSAB containing cefoperazone (2 μg/mL), vancomycin (10 μg/mL), and amphotericin B (2 μg/mL) (all antibiotics were obtained from Sigma-Aldrich, St. Louis MO) agar plates and grown in anaerobic jars equilibrated with CampyGen sachets (Oxoid) at 37 °C for 48–72 h. The resulting growth was assigned a score on a scale of 0–4 based on the density of growth; 0 (no growth), 1 (1–20 CFU), 2 (20–200 CFU), 3 (200–400 CFU), and 4 (confluent growth) as described [30].

### Neurological phenotyping

Starting 1 week before experimental infections and then daily after inoculation with a *C. jejuni* strain, mice were observed daily for evidence of enteric and neurological disease. Daily monitoring was based on previously published clinical exam score sheets designed to score feature of gastrointestinal and neurological signs [30, 33]. Additionally, open-field testing was performed to detect neurological signs and changes in behavior due to inoculation with either GBS-associated or enteric-associated strains of *C. jejuni*. All TSB sham-inoculated control mice served as controls for phenotyping. The activity of all experimental mice was video-recorded once per week for 1 week before inoculation and once per week for 7 weeks post-inoculation. Briefly, mice were placed in the center of an 18′′ × 8′′ sterile rat cage divided into four marked quadrants and allowed to move freely for 90 s. At the completion of the experiment, a single investigator (PTB), who was blinded to mouse group identity, recorded the number of quadrants crossed and the number of rears for each mouse. Quadrants crossed were counted starting with the first line crossed after establishing all four limbs in a single quadrant. Rears were counted as the extension of hind limbs and placement of both front limbs on the side of the cage.

### Scoring of ileocecocolic junction histopathology

Tissue samples were collected at necropsy, placed in cassettes, fixed in 10% NBF (Fisher Scientific) for 20–24 h, and then transferred to 60% ethanol until final processing. Samples were submitted to the Michigan State University Investigative Histopathology Laboratory where they were processed in the following manner: fixed samples were vacuum-infiltrated with paraffin on the Sakura VIP 2000 tissue processor; followed by embedding with a ThermoFisher HistoCentre III embedding station. Paraffin-embedded blocks were sectioned at 4–5 μm with a rotary microtome, dried at 56 °C in a slide incubator for 2–24 h, and stained with Hematoxylin and Eosin (H&E). Scoring of the distal ileum, cecum, and proximal colon was performed as described [30]. Briefly, the lumen, epithelium, lamina propria, and submucosa of the ileocecocolic junction (ICCJ) of each mouse were observed for histopathological changes by a single investigator (LSM) blinded to sample identity, and a score from 1 to 41 was assigned based on lesions using a standardized scoring system. Specific features evaluated among others were as follows: (1) excess mucus and inflammatory exudates in the lumen; (2) surface integrity, intraepithelial lymphocyte number, goblet cell hypertrophy, goblet cell depletion, crypt hyperplasia, crypt atrophy, crypt adenomatous changes, and crypt inflammation in the epithelium; (3) increased immune cells in the lamina propria; (4) and fibrosis in the submucosa.

### Cytokine analysis

RNA was extracted from proximal colon samples that were flash frozen at the time of necropsy. Equal sized 5-mm-cubed tissue snips were homogenized using micropestles, and RNA was extracted following the RNeasy Plus Mini Kit protocol (QIAGEN). RNA concentrations were measured using the Nanodrop ND-1000 spectrophotometer and standardized to a concentration of 50 ng/μL. cDNA was obtained by PCR with random primers. A master mix was assembled using reagents from Promega GoTaq qPCR kit and added to the samples. This reaction was run using the following thermal cycler conditions: step 1, 5 min 25 °C; step 2, 20 min 42 °C; step 3, 70 °C; and step 4, 4 °C min—Hold. Interleukin 4 (IL-4) and Interferon gamma (IFNγ) cytokine levels were measured using qPCR on an iQ5 thermocycler (Bio-Rad) with standardization. ANOVAs were performed on 2-ΔΔct data to find the linear fold change in gene expression and are presented as mean fold change of three replicates over levels of the housekeeping gene hypoxanthine-guanine phosphoribosyltransferase (HPRT).

### Enzyme-linked immunosorbent assays

Indirect enzyme-linked immunosorbent assays (ELISAs) were performed to test for the presence of antibodies reactive with bulk *C. jejuni* antigen and/or gangliosides GM1, GD1a, and GQb1 in the plasma of experimental mice, referred to as anti-*Campylobacter* and anti-ganglioside antibodies, respectively. Preparation of the bulk *C. jejuni* antigen was performed as previously described [30, 35]. Positive controls (highly reactive plasma samples that tested strongly for the presence of the antigen in previous experiments) and negative controls (monoclonal mouse anti-*Toxoplasma gondii*, ViroStat) were used in all cases. All samples were run in triplicate and the mean values used for statistical analysis. We tested for antibodies to gangliosides GM1 (Sigma), GD1a (USBio), and mixed GM1-GQ1b (Sigma, Calbiochem, respectively) [33]. Immunoglobulin (IgG) subtypes were determined using biotinylated goat anti-mouse-IgG1, IgG2b, IgG2c, and IgG3 (Jackson ImmunoResearch, West Grove, PA) secondary antibodies. Methods for *C. jejuni*-specific antibody ELISAs were described previously [30] and ganglioside ELISAs were conducted similarly [33].

### Quantification of F4/80 positive cells in sciatic nerves and dorsal root ganglia

Sciatic nerves and 2–3 lumbar dorsal root ganglia (DRG) from L3, L4, and L5 were dissected, isolated, and fixed in 10% formalin pH 7.0. After that, tissues were embedded en bloc in order to assess the segmental nature of any GBS lesions [33]. Slides were prepared by the Michigan State University Investigative Histopathology Laboratory. Briefly, 3–5 μm sections were placed on charged slides, dried at 56 °C for approximately 12 h, and subsequently deparaffinized in xylene and hydrated through descending grades of ethyl alcohol to distilled water. Slides were placed in Tris-buffered saline (TBS) pH 7.4 (Scytek Labs—Logan, UT) for 5 min for pH adjustment. Following TBS, epitope retrieval was performed using Citrate Plus Retrieval Solution pH 6.0 (Scytek) in a vegetable steamer for 30 min followed by a 10-min countertop incubation and several changes of distilled water. Following pretreatment standard, avidin-biotin complex staining steps were performed at room temperature on the DAKO Autostainer. All staining steps are followed by 2-min rinses in Tris-buffered saline and Tween 20 (Scytek). After blocking with Normal Rabbit Serum (Vector Labs—Burlingame, CA) for 30 min, sections were incubated with avidin-biotin blocking system for 15 min each (Avidin D—Vector Labs/d-Biotin—Sigma). Primary antibody slides were incubated for 60 min with the Monoclonal Rat anti-Mouse F4/80 diluted at 1:100 (AbD Serotec—Raleigh, NC) in normal antibody diluent (NAD) (Scytek). Reaction development utilized Vector Nova Red Kit peroxidase chromogen incubation of 15 min followed by counterstaining in Gill’s Hematoxylin (Cancer Diagnostics—Durham, NC) for 30 s, differentiated with 1% acetic acid, dehydrated, and mounted with Permount (Sigma). F4/80 stained cells were counted and normalized for tissue area using ImageJ version 2.0.0-rc43/1.50e [51].

### Statistical analysis

Statistical analyses were performed using GraphPad Prism 6.0 h for Mac OS X (GraphPad Software, La Jolla, California USA) with the exception of 16S rRNA gene amplicon analysis. Data were entered and then checked for normality and equal variance. If they passed both tests, one-way ANOVA was performed. If they failed either test, a Kruskal-Wallis test was performed instead, followed by Dunn’s post-test, with *P* < 0.05 constituting significance. Statistical analysis of histopathological scoring of ICCJ was performed using a Kruskal-Wallis test followed by Dunn’s post-test. Statistically significant comparisons in histopathology were further analyzed using Fisher’s exact test (http://vassarstats.net/fisher2x3.html) and corrected for multiple comparisons with the Holm-Sidak step-down procedure [30]. Two-way repeated measures ANOVA and Tukey’s post-test were used for analysis of open-field and rearing behavior in Experiment 1. Analysis of 16S rRNA gene amplicon data was performed using PAST 3 [50]; statistical procedures are indicated in figure legends.

For comparison of anti-ganglioside antibody levels between experimental groups, all datasets had unequal variances by one-way ANOVA so Kruskal-Wallis test was used in PAST. If the full table had a significant *P* in the Kruskal-Wallis test, pairwise tests between groups were conducted using the Mann-Whitney test; *P* values were adjusted for multiple comparisons using the Bonferroni procedure.

## Additional files

Additional file 1: Figure S1. Handling techniques do not alter abundance of select OTUs. (PPTX 151 kb)

Additional file 2: Table S1. Principle components analysis. (PDF 82 kb)

Additional file 3: Table S2. Indicator groups. Assemblage of indicator OTUs detected using indicator from the mothur pipeline. (PDF 179 kb)

Additional file 4: Python code used to generate heatmap for 60 most abundant OTUs in humanized and conventional mice. OTUs were sorted, the 60 most abundant OTUs retrieved, and those OTUs clustered according to relative abundance using python (v 3.5) and seaborn (version 0.7.1). OTU abundance increases from light green to dark green. Left y-axis labels (network_pal) corresponds to the microbiota or inoculum; Humanized =brown; Conventional = bluegreen; inoculum = Teal. Right y-axis labels represent individual samples starting with group labels. Group labels; HI2= Humanized-Infected-260.94, HUT= Humanized-Uninfected-TSB, HI1= Humanized-Infected-11168, CI2= Conventional-Infected-260.94, CUT= Conventional-Uninfected-TSB, CI1= Conventional-Infected-11168, INO= Inoculum. (MD 4 kb)

Additional file 5: SraRun Table. NCBI SRA sample submission report for bioproject PRJNA380673. (TXT 18 kb)

Additional file 6: Relative abundances for all operational taxonomic units. Abundances of OTUs were determined using mothur. (CSV 41 kb)

Additional file 7: Taxonomic assignments for all OTUs. Taxonomic assignments for OTUs in Additional file 3 determined using mothur. Reads were binned into OTUs using cluster.split and the average neighbor algorithm (cutoff=0.03). (CSV 20 kb)

## Abbreviations

^Conv^microbiota: Conventional microbiota
DRG: Dorsal root ganglia
ELISAs: Enzyme-linked immunosorbent assays
GBS: Guillain Barré syndrome
H&E: Hematoxylin and eosin
HPRT: Hypoxanthine-guanine phosphoribosyl transferase
^Hu^microbiota: Human microbiota
ICCJ: Ileocecocolic junction
IgG: Immunoglobulin G
IL-10: Interleukin-10
NAD: Nicotinamide adenine dinucleotide
NBF: Neutral-buffered formalin
OTU: Operational taxonomic units
PI: Post infection
SPF: Specific pathogen free
TBS: Tris-buffered saline
T_H_1: T helper 1
T_H_2: T helper 2
TSB: Tryptose soy broth
