# Supplementary figures and images for: Transplanted human fecal microbiota enhanced Guillain Barré syndrome autoantibody responses after Campylobacter jejuni infection in C57BL/6 mice
Source: Microbiome. 2017 Aug 8;5:92. doi: 10.1186/s40168-017-0284-4 (PMC5547673; doi:10.1186/s40168-017-0284-4)

## Slide 1
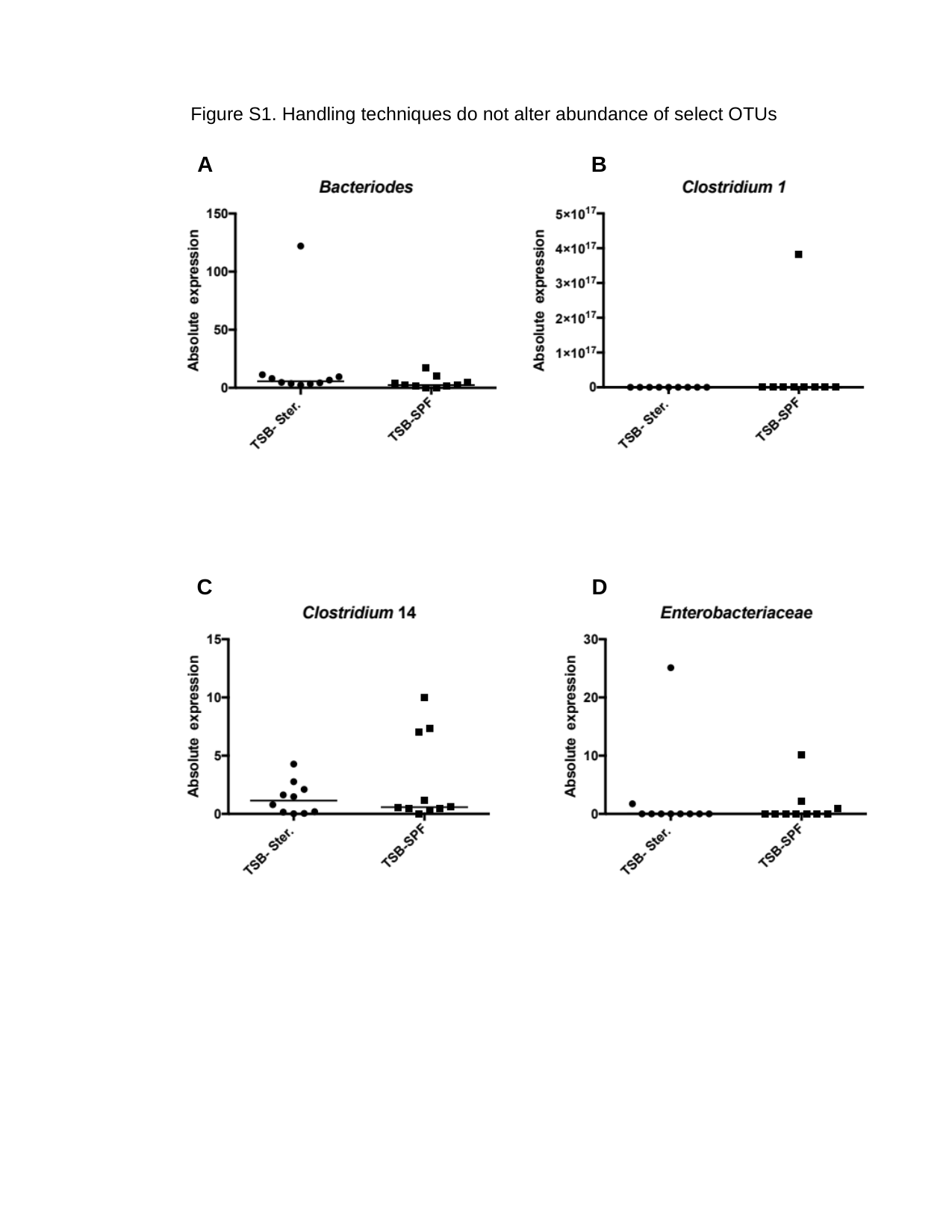

Figure S1. Handling techniques do not alter abundance of select OTUs
A
B
C
D

Supplement: Supplementary file 1 — Handling techniques do not alter abundance of select OTUs. (PPTX 151 kb) [file 40168_2017_284_MOESM1_ESM.pptx]
