# Supplementary material for: Transplanted human fecal microbiota enhanced Guillain Barré syndrome autoantibody responses after Campylobacter jejuni infection in C57BL/6 mice
Source: Microbiome. 2017 Aug 8;5:92. doi: 10.1186/s40168-017-0284-4 (PMC5547673; doi:10.1186/s40168-017-0284-4)
Supplement: Supplementary file 2 — Principle components analysis. (PDF 82 kb) [file 40168_2017_284_MOESM2_ESM.pdf]

Table S1

PC	Eigenvalue	% variance	cumulative % variance	Loadings	Axis 1	Axis 2	Axis 3	Axis 4
1	1.94E+06	62.851	62.851	Otu001	-0.2182	-0.8801	0.2965	0.1682
2	826530	26.76	89.611	Otu002	0.706	0.1061	0.6459	0.02955
3	127212	4.1187	93.7297	Otu003	-0.6125	0.3932	0.5477	0.158
4	55634.1	1.8013	95.531	Otu004	0.01291	0.02193	0.002615	-0.03965
5	30221.9	0.97849	96.50949	Otu005	0.06274	-0.03756	-0.1443	-0.01264
6	21630.2	0.70032	97.20981	Otu006	-0.1501	0.1762	-0.04845	-0.08714
7	18795.2	0.60853	97.81834	Otu007	0.0673	-0.02801	-0.0813	-0.0107
8	16583.8	0.53693	98.35527	Otu008	-0.1116	0.05497	0.09157	0.02544
9	11358.6	0.36776	98.72303	Otu009	-0.00615	-0.00233	-0.09456	-0.03379
10	7004.58	0.22679	98.94982	Otu010	0.02819	-0.00446	-0.0382	0.03239
11	6379.05	0.20653	99.15635	Otu011	0.1294	0.1127	-0.2546	0.8871
12	5644.54	0.18275	99.3391	Otu012	0.07659	0.01339	0.004896	-0.08691
13	5286.03	0.17115	99.51025	Otu013	-0.06895	0.06937	-0.0118	-0.03991
14	3213.42	0.10404	99.61429	Otu014	0.01091	-0.0029	0.01926	-0.01026
15	2542.71	0.082325	99.696615	Otu015	0.004843	0.01695	-0.02093	0.02365
16	1972.54	0.063865	99.76048	Otu016	-0.02603	-0.01537	0.02613	-0.00365
17	1326.41	0.042945	99.803425	Otu017	-0.00166	-0.00312	-0.1796	-0.2537
18	1147.25	0.037144	99.840569	Otu018	-0.02324	0.0312	-0.00296	0.01637
19	851.783	0.027578	99.868147	Otu019	-0.0122	0.01178	0.007099	0.000479
20	821.662	0.026603	99.89475	Otu020	-0.00715	0.005757	0.005393	-0.00108
21	714.81	0.023143	99.917893	Otu021	0.000594	0.001608	-0.00678	-0.00192
22	531.086	0.017195	99.935088	Otu022	0.001861	0.000195	0.000169	-0.00165
23	399.055	0.01292	99.948008	Otu023	0.02089	0.004615	-0.07937	-0.09514
24	313.592	0.010153	99.958161	Otu024	0.000379	-0.01701	-0.00596	0.005465
25	253.457	0.0082061	99.9663671	Otu025	-0.00572	0.008306	-0.00841	-0.0058
26	235.21	0.0076154	99.9739825	Otu026	0.02676	0.02092	-0.122	0.06369
27	174.885	0.0056622	99.9796447	Otu027	0.004766	0.000971	-0.01679	0.01258
28	159.849	0.0051754	99.9848201	Otu028	0.00056	-0.00132	-0.02285	-0.03172
29	118.175	0.0038261	99.9886462	Otu029	0.001761	0.000487	0.001905	-0.00521
30	96.3741	0.0031203	99.9917665	Otu030	0.03391	0.00089	-0.02664	-0.00729
31	42.9081	0.0013892	99.9931557	Otu031	-8.32E-05	-0.00169	-0.05683	-0.08048
32	36.1385	0.0011701	99.9943258	Otu032	0.01342	-0.02532	-0.04394	-0.00301
33	29.7293	0.00096254	99.99528834	Otu033	0.000225	0.000351	-0.00078	-0.00163
34	23.693	0.00076711	99.99605545	Otu034	-0.00086	-0.00097	-0.07109	-0.09847
35	21.9022	0.00070913	99.99676458	Otu035	-0.00674	-0.00626	-0.03671	-0.07077
36	18.3659	0.00059463	99.99735921	Otu036	-0.00031	-0.0009	-0.02673	-0.03871
37	15.2968	0.00049526	99.99785447	Otu037	-0.00654	0.007456	-0.00175	-0.00241
38	13.6742	0.00044273	99.9982972	Otu038	-0.00231	-0.00177	0.00194	-0.00159
39	6.90225	0.00022347	99.99852067	Otu039	0.019	-0.04003	-0.07648	0.005851
40	5.5542	0.00017983	99.9987005	Otu040	0.04777	0.0138	0.03961	-0.1656
41	4.07813	0.00013204	99.99883254	Otu041	0.000857	0.000242	0.000796	0.001377
42	3.36897	0.00010908	99.99894162	Otu042	-0.00343	0.00489	-0.01004	0.000698
43	2.59045	8.39E-05	99.99902549	Otu043	-8.14E-05	-3.01E-06	-0.00283	-0.00401
44	2.19957	7.12E-05	99.99909671	Otu044	-0.00013	0.00053	-0.00279	-0.00184
45	1.66254	5.38E-05	99.99915053	Otu045	-6.77E-05	-0.00063	-0.02233	-0.03157
46	1.35688	4.39E-05	99.99919447	Otu046	0.000675	9.55E-05	-0.00075	-0.00167
47	1.14992	3.72E-05	99.9992317	Otu047	0.0019	-0.00322	-0.0095	0.001473
48	0.881844	2.86E-05	99.99926025	Otu048	0.000154	3.54E-05	-0.00059	-0.00107
49	0.681398	2.21E-05	99.99928231	Otu049	-0.00891	0.000638	0.01921	0.008613
50	0.562397	1.82E-05	99.99930052	Otu050	0.001456	0.000223	-0.00026	-0.00119
51	0.424325	1.37E-05	99.99931426	Otu051	0.002058	-0.00233	-0.00192	0.004534

Table S1 Continued

PC	Eigenvalue	% variance	cumulative % variance
52	0.326128	1.06E-05	99.99932482
53	0.291016	9.42E-06	99.99933424
54	0.211521	6.85E-06	99.99934109
55	0.139693	4.52E-06	99.99934561
56	0.120737	3.91E-06	99.99934952
57	0.0998211	3.23E-06	99.99935275
58	0.0692759	2.24E-06	99.99935499
59	0.0578644	1.87E-06	99.99935687
60	0.0369007	1.19E-06	99.99935806

Loadings	Axis 1	Axis 2	Axis 3	Axis 4
Otu052	-9.62E-06	-0.0005	-0.01709	-0.02434
Otu053	0.001549	-0.00197	-0.00542	-0.00329
Otu054	-0.00022	-0.00036	-0.02071	-0.0301
Otu055	-2.17E-05	-0.00036	-0.01284	-0.01825
Otu056	-0.0044	-0.00153	0.005415	0.005099
Otu057	-0.00027	0.000196	0.000116	-0.00043
Otu058	-0.0001	3.14E-05	0.000138	-4.03E-05
Otu059	-0.00182	0.00427	-0.03201	-0.02662
Otu060	0.000363	-0.0012	-0.00081	-0.00042
Otu061	-2.91E-05	-0.00044	-0.01801	-0.02557
Otu062	-0.00012	-0.00037	-0.01384	-0.02001
Otu063	-0.00059	0.000793	0.000125	-0.0016
Otu064	-7.34E-05	9.96E-05	-0.00013	0.000132
Otu065	-0.00045	0.000614	-0.00207	-0.00289
Otu066	-3.88E-07	-4.23E-06	-0.00017	-0.00025
Otu067	-1.59E-05	-1.43E-05	-7.18E-05	-0.00015
Otu068	-3.14E-05	-0.00085	-0.0352	-0.05115
Otu069	1.65E-05	-1.55E-05	-8.00E-05	4.78E-06
Otu070	6.05E-05	2.19E-05	7.63E-05	0.000164
Otu071	-2.16E-05	3.76E-05	-4.14E-05	6.33E-05
Otu072	2.06E-06	1.91E-05	-0.00013	-0.00029
Otu073	0.000486	-0.00084	-0.00185	0.001664
Otu074	-0.00041	0.000506	-0.00482	-0.00567
Otu075	0.000224	-0.00114	-0.00312	0.00151
Otu076	-8.37E-06	1.94E-05	-6.48E-05	-2.64E-07
Otu077	5.08E-06	-6.42E-05	-0.0008	-0.00107
Otu078	-8.29E-05	6.40E-05	0.000166	4.18E-05
Otu079	-0.00023	0.000266	-5.38E-05	-2.53E-05
Otu080	-1.26E-05	-0.0002	-0.00721	-0.01024
Otu081	7.85E-05	5.98E-05	-6.69E-05	0.000856
Otu082	-1.94E-06	-2.12E-05	-0.00087	-0.00125
Otu083	0.00038	-0.00069	-0.00064	0.001104
Otu084	-2.33E-06	-2.54E-05	-0.00105	-0.00149
Otu085	6.89E-05	-0.00026	-0.00094	0.000539
Otu086	5.22E-05	1.35E-05	-0.00064	-0.00019
Otu087	-7.67E-05	-4.89E-05	9.77E-05	-9.33E-05
Otu088	-2.33E-06	-2.54E-05	-0.00105	-0.00149
Otu089	-3.99E-07	1.21E-05	-9.02E-06	0.00013
Otu090	-3.08E-06	-4.43E-05	-5.68E-05	1.66E-05
Otu091	6.77E-05	-0.00022	-0.00072	0.000823
Otu092	0.000142	-0.00011	-0.00043	0.000149
Otu094	-3.88E-07	-4.23E-06	-0.00017	-0.00025
Otu095	9.30E-05	3.07E-05	-0.00031	0.000696
Otu096	4.10E-05	-7.49E-05	-5.83E-06	0.000221
Otu097	-1.16E-05	1.88E-05	-0.00018	-0.00019
Otu098	1.30E-05	5.04E-06	-1.33E-05	-2.08E-06
Otu100	-1.11E-05	1.25E-05	-0.00019	-0.00034
Otu101	-1.13E-05	-7.86E-06	1.99E-05	-1.99E-05
Otu103	1.44E-05	-8.52E-08	4.18E-05	-0.00012
Otu104	1.27E-05	2.06E-06	-4.00E-06	1.29E-05
Otu105	6.74E-06	2.98E-05	-6.71E-05	0.000157
Otu106	2.08E-06	-2.50E-05	-6.27E-05	1.93E-05

Table S1 continued

PC	Eigenvalue	% variance	cumulative % variance	Loadings	Axis 1	Axis 2	Axis 3	Axis 4
				Otu107	6.10E-05	1.08E-05	-9.04E-05	0.00027
				Otu108	-3.88E-07	-4.23E-06	-0.00017	-0.00025
				Otu109	3.67E-05	1.97E-05	-0.00013	0.00017
				Otu112	-1.11E-05	3.25E-06	1.80E-06	-5.05E-05
				Otu113	1.59E-05	-3.21E-05	1.13E-05	-1.37E-05
				Otu114	-3.88E-07	-4.23E-06	-0.00017	-0.00025
				Otu116	1.12E-05	-5.42E-06	-0.0002	-0.00012
				Otu117	1.04E-05	3.45E-06	-6.79E-05	8.37E-05
				Otu118	7.85E-05	5.98E-05	-6.69E-05	0.000856
				Otu119	9.43E-05	7.17E-05	-8.02E-05	0.001027
				Otu121	3.14E-05	2.39E-05	-2.67E-05	0.000342
				Otu123	2.93E-06	-2.79E-05	-0.00022	-0.00029
				Otu124	-3.88E-07	-4.23E-06	-0.00017	-0.00025
				Otu125	5.32E-06	-7.31E-05	2.23E-05	-2.92E-05
				Otu126	-3.88E-07	-4.23E-06	-0.00017	-0.00025
				Otu127	3.14E-05	2.39E-05	-2.67E-05	0.000342
				Otu130	-3.88E-07	-4.23E-06	-0.00017	-0.00025
				Otu132	5.85E-06	-5.57E-05	-0.00045	-0.00057
				Otu135	-8.37E-06	1.94E-05	-6.48E-05	-2.64E-07
				Otu139	1.15E-05	-1.19E-06	-2.13E-05	0.000128
				Otu140	3.33E-05	-6.87E-05	8.04E-05	-5.15E-05
				Otu144	-3.51E-06	-9.87E-05	8.54E-05	-3.83E-05
				Otu149	5.06E-05	1.72E-05	-8.62E-05	0.000226
				Otu164	2.54E-05	4.12E-06	-8.00E-06	2.58E-05
				Otu178	1.08E-05	-4.94E-07	-3.72E-05	9.57E-05
