# Supplementary material for: Transplanted human fecal microbiota enhanced Guillain Barré syndrome autoantibody responses after Campylobacter jejuni infection in C57BL/6 mice
Source: Microbiome. 2017 Aug 8;5:92. doi: 10.1186/s40168-017-0284-4 (PMC5547673; doi:10.1186/s40168-017-0284-4)
Supplement: Supplementary file 3 — Indicator groups. Assemblage of indicator OTUs detected using indicator from the mothur pipeline. (PDF 179 kb) [file 40168_2017_284_MOESM3_ESM.pdf]

Table S2 - Indicator groups identified by mothur

	Phylum	Class	Order	Family	Genus	P value
**Conventional microbiota mice**						
Otu002	*Bacteroidetes*	*Bacteroidia*	*Bacteroidales*	*Porphyromonadaceae*	unclassified	0.024
Otu005	*Firmicutes*	*Clostridia*	*Clostridiales*	unclassified	unclassified	0.048
Otu012	*Firmicutes*	*Bacilli*	*Lactobacillales*	*Lactobacillaceae*	*Lactobacillus*	0.008
Otu022	*Firmicutes*	*Bacilli*	*Lactobacillales*	unclassified	unclassified	<0.001000
Otu029	*Firmicutes*	*Bacilli*	*Lactobacillales*	*Lactobacillaceae*	unclassified	0.016
Otu032	*Firmicutes*	*Clostridia*	unclassified	unclassified	unclassified	0.024
Otu039	*Tenericutes*	*Mollicutes*	*Anaeroplasmatales*	*Anaeroplasmataceae*	*Anaeroplasma*	0.032
Otu040	*Firmicutes*	*Erysipelotrichia*	*Erysipelotrichales*	*Erysipelotrichaceae*	*Allobaculum*	0.032
Otu041	*Firmicutes*	*Bacilli*	unclassified	unclassified	unclassified	<0.001000
Otu047	*Firmicutes*	*Clostridia*	*Clostridiales*	*Lachnospiraceae*	*Clostridium_XlVb*	0.024
**Humanized microbiota mice**						
Otu003	*Bacteroidetes*	*Bacteroidia*	*Bacteroidales*	*Bacteroidaceae*	*Bacteroides*	0.032
Otu008	*Bacteroidetes*	*Bacteroidia*	*Bacteroidales*	*Porphyromonadaceae*	*Parabacteroides*	0.048
Otu013	*Bacteroidetes*	*Bacteroidia*	*Bacteroidales*	*Rikenellaceae*	*Alistipes*	0.048
Otu019	*Proteobacteria*	*Betaproteobacteria*	*Burkholderiales*	*Sutterellaceae*	*Sutterella*	0.016
Otu025	*Proteobacteria*	*Betaproteobacteria*	unclassified	unclassified	unclassified	0.032
Otu049	*Proteobacteria*	*Epsilonproteobacteria*	*Campylobacterales*	*Campylobacteraceae*	*Campylobacter*	0.04
Otu079	*Proteobacteria*	*Betaproteobacteria*	*Burkholderiales*	*Oxalobacteraceae*	unclassified	0.008
